# Global research trends in tryptophan metabolism and cancer: a bibliometric and visualization analysis (2005–2024)

**DOI:** 10.3389/fonc.2025.1621666

**Published:** 2025-07-01

**Authors:** Huanhuan Ma, Ran Ding, Junwen Wang, Guangying Du, Yun Zhang, Qiuchen Lu, Yingyue Hou, Haosong Chen, Hongguan Jiao

**Affiliations:** ^1^ School of Information Engineering, Guizhou University of Traditional Chinese Medicine, Guiyang, China; ^2^ College of Basic Traditional Chinese Medicine, Guizhou University of Traditional Chinese Medicine, Guiyang, China; ^3^ Institute of Basic Theory of Traditional Chinese Medicine, China Academy of Chinese Medical Sciences, Beijing, China

**Keywords:** tryptophan metabolism, cancer, kynurenine, TME, bibliometrics, visualization

## Abstract

**Background:**

In recent years, tryptophan metabolism has gained increasing attention for its pivotal role in shaping the tumor immune microenvironment and promoting cancer progression. As a result, it has become a central topic in cancer metabolism and tumor immunology. This study applies a comprehensive bibliometric approach to analyze global research trends in tryptophan metabolism within the context of cancer. By identifying emerging hotspots, leading contributors, and patterns of international collaboration, this work aims to provide meaningful insights to guide future therapeutic strategies targeting metabolic pathways in oncology.

**Methods:**

A systematic literature search was performed using the Web of Science Core Collection to retrieve publications related to tryptophan metabolism in cancer from 2005 to 2024. Bibliometric and visual analyses were conducted using CiteSpace, VOSviewer, and Python to examine publication trends, national and institutional contributions, author productivity, journal influence, co-citation networks, and keyword co-occurrence patterns.

**Results:**

A total of 1,927 publications were identified, authored by 11,134 researchers from 70 countries and published in 781 academic journals. The volume of publications showed a steady increase, peaking in 2021. The United States and China emerged as the dominant contributors, excelling in both research output and international collaboration. Dietmar Fuchs was identified as the most prolific author, with 61 publications. The Medical University of Innsbruck was the leading institution, with 144 publications. *Frontiers in Immunology* demonstrated strong citation performance and academic impact. Co-citation and keyword analysis revealed key research themes, including “IDO (indoleamine 2,3-dioxygenase),” “tryptophan catabolism,” “cancer,” and “dendritic cells,” as well as emerging topics such as “gut microbiota,” “tumor microenvironment,” “aryl hydrocarbon receptor,” and “cancer immunotherapy.”

**Conclusion:**

This study highlights the growing significance of tryptophan metabolism research in cancer, underlining the complex interactions between metabolic pathways and immune responses. Further investigations are needed to explore the therapeutic potential of these metabolic pathways, which could lead to novel cancer treatment strategies.

## 1 Introduction

Cancer is a highly complex and heterogeneous disease characterized by the dysregulation of normal cellular control mechanisms, leading to the abnormal proliferation and dissemination of malignant cells (1). This uncontrolled growth threatens patients’ physical and mental well-being and adversely affects family dynamics and interpersonal relationships. According to the World Health Organization (WHO), cancer is the second leading cause of death worldwide after cardiovascular diseases, causing approximately 10 million deaths annually. Projections indicate that from 2020 to 2050, the global economic burden of cancer will reach $25.2 trillion, equivalent to an average annual tax burden of 0.55% of the global gross domestic product (GDP) (2). By 2050, the global incidence of cancer is projected to rise to 35.3 million new cases—a 76.6% increase—while cancer-related mortality may reach 18.5 million, representing an 89.7% increase (3). Despite advances in conventional treatments such as surgery, chemotherapy, and radiotherapy, drug resistance remains a major unresolved challenge. To overcome this, modern approaches including targeted therapy, immunotherapy, gene therapy, stem cell therapy, natural antioxidants, photodynamic therapy, nanoparticles, and precision medicine are being applied to cancer diagnosis and treatment (4–8). Among these, metabolic therapy has attracted considerable interest for its promising therapeutic potential (9).

Drug resistance primarily arises from tumors establishing compensatory signaling pathways, alterations in target proteins, changes in the tumor microenvironment, tumor heterogeneity, and adaptation to targeted therapies. The interaction of these factors drives the development of acquired resistance to targeted treatments (10). To date, the U.S. Food and Drug Administration (FDA) has approved drugs targeting over 30 distinct molecular targets (11–13), offering new hope to patients. Advances in technologies such as whole-genome sequencing, targeted high-throughput sequencing, and deep sequencing have enabled the detection of aberrant tumor genes with greater precision. Immunotherapies—including immune checkpoint inhibitors, CAR-T cell therapies, and cancer vaccines (14, 15) —leverage the host immune system to selectively eliminate malignant cells while sparing normal tissues (16). Notably, PD-1/PD-L1 antibody therapies have shown remarkable efficacy and durable responses across various cancers, with fewer side effects than conventional treatments (17, 18).

Studies have demonstrated that tumor cells preferentially metabolize glucose into lactate via glycolysis even in the presence of oxygen, a phenomenon known as the Warburg effect. This metabolic reprogramming provides a theoretical foundation for tumor metabolic therapy (19). Among metabolic pathways, tryptophan metabolism has attracted significant attention due to its essential role in regulating inflammation, metabolism, immune responses, and neurological functions (20). Beyond these physiological processes, tryptophan metabolism has been implicated in tumor progression by suppressing anti-tumor immunity and promoting tumor cell malignancy (21). In particular, enzymes involved in the kynurenine pathway—such as IDO and tryptophan 2,3-dioxygenase (TDO)—have been correlated with poor prognosis in multiple cancer types (22).

**Figure 1:**
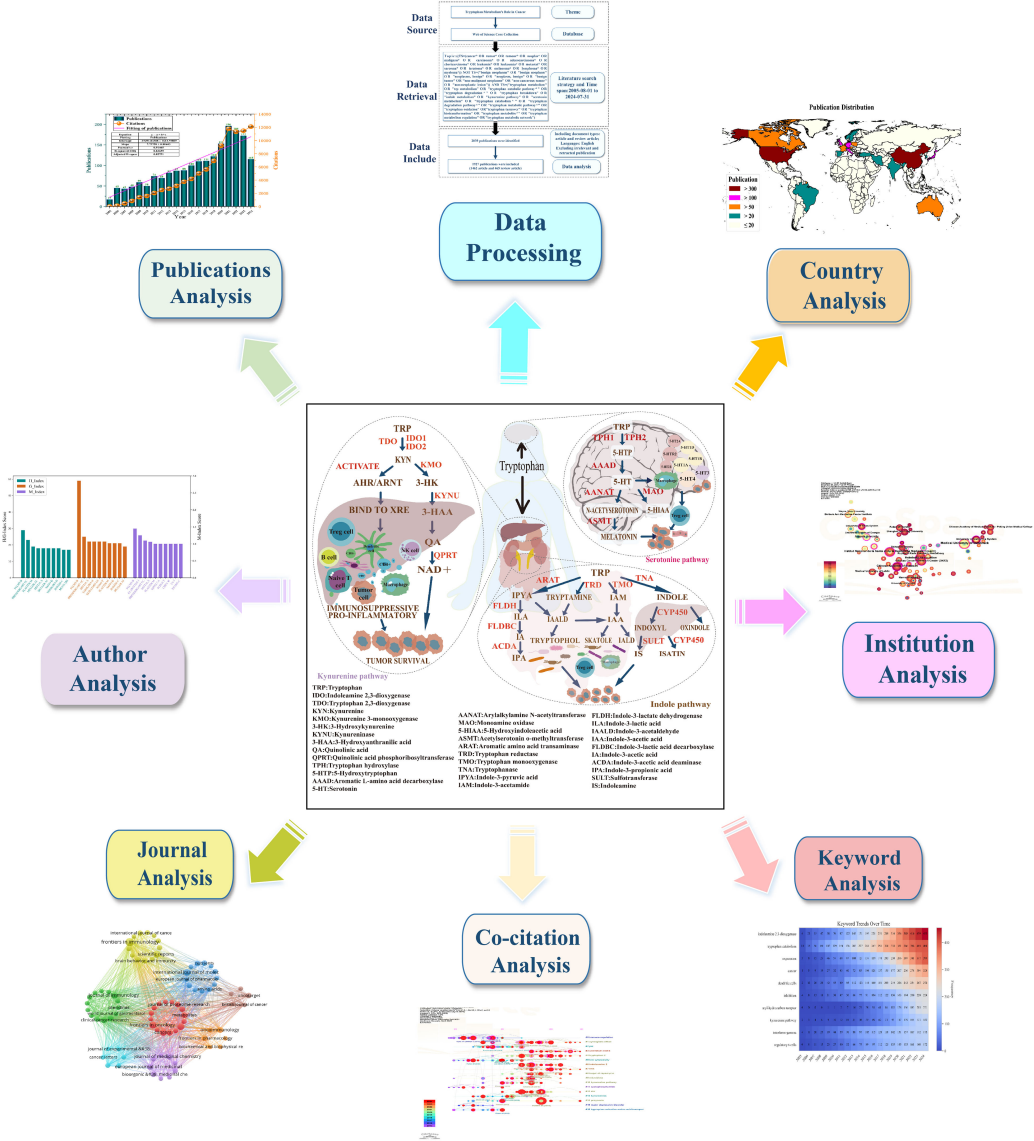
Graphical abstract illustrating the bibliometric analysis of tryptophan metabolism in cancer. Publications related to tryptophan metabolism in cancer were retrieved from the Web of Science Core Collection (WOSCC). Visualization analyses were conducted using multiple tools, covering various perspectives including annual publication trends, leading countries, prolific authors, influential institutions, journals, co-cited references, and keyword co-occurrence.

As research on tryptophan metabolism in cancer advances, there is an increasing need to explore the current status and emerging trends in this field. Bibliometrics has become a powerful and widely used tool for analyzing and evaluating scientific literature across disciplines. As an interdisciplinary science, bibliometrics employs mathematical and statistical methods alongside visualization techniques to quantitatively analyze large bodies of literature within specific research domains, thereby revealing patterns and trends in the development of scientific topics (23). This quantitative approach offers an objective and intuitive assessment of past academic activities and achievements, minimizing potential biases arising from subjective evaluation (24). By quantifying research output, bibliometrics enables comparisons of scholarly productivity across countries, institutions, authors, and journals, and facilitates the identification of cutting-edge research and publication trends (25). Although bibliometric analyses have been widely conducted in many fields, no comprehensive bibliometric study has yet addressed tryptophan metabolism in cancer. Therefore, this study employs bibliometric analysis to comprehensively examine the literature on tryptophan metabolism in cancer from 2005 to 2024. It aims to identify the developmental trajectory, current research landscape, and emerging trends, while providing guidance for future research directions and therapeutic strategies (see **Figure 1**).

## 2 Materials and methods

### 2.1 Data acquisition and search strategy

The Web of Science is globally recognized as a leading authoritative database, indexing a wide range of high-quality academic journals, conference papers, books, patents, and other scholarly works. We conducted a literature search in the Web of Science Core Collection for documents related to tryptophan metabolism and cancer, utilizing the following search strategy: Topic: ((TS=(cancer* OR tumor* OR tumour* OR neoplas* OR malignan* OR carcinoma* OR adenocarcinoma* OR choricarcinoma* OR leukemia* OR leukaemia* OR metastat* OR sarcoma* OR teratoma* OR melanoma* OR lymphoma* OR myeloma*)) NOT TS=(“benign neoplasms” OR “benign neoplasm” OR “neoplasms, benign” OR “neoplasm, benign” OR “benign tumor” OR “non-malignant neoplasms” OR “non-cancerous tumor” OR “non-neoplastic lesion”)) AND TS=(“tryptophan metabolism” OR “trp metabolism” OR “tryptophan catabolic pathway*” OR “tryptophan degradation*” OR “tryptophan breakdown” OR “indole metabolism” OR “kynurenine pathway” OR “serotonin metabolism” OR “tryptophan catabolism*” OR “tryptophan degradation pathway*” OR “tryptophan metabolic pathway*” OR “tryptophan oxidation” OR “tryptophan turnover” OR “tryptophan biotransformation” OR “tryptophan metabolite*” OR “tryptophan metabolism regulation” OR “tryptophan metabolic network”).

### 2.2 Inclusion and exclusion criteria

Preliminary searches revealed that the earliest publication addressing the role of tryptophan metabolism in cancer development dates back to 1955, marking the initial exploration of this field. From 1955 to 2004, a total of 313 relevant publications were retrieved, accounting for 13.3% of the overall dataset (2,352 articles). However, the annual publication output during this period remained low, with most years recording fewer than ten articles, and only 20 articles published in 2004. These findings suggest that, although early research laid a foundational basis, the field was still in its infancy and lacked sustained and systematic development. In contrast, the period from 2005 to 2024 witnessed a significant acceleration in research activity, characterized by a steady increase in annual publication volume. A total of 2,039 articles were retrieved during this stage, indicating greater academic interest and a more robust research output.

**Figure 2:**
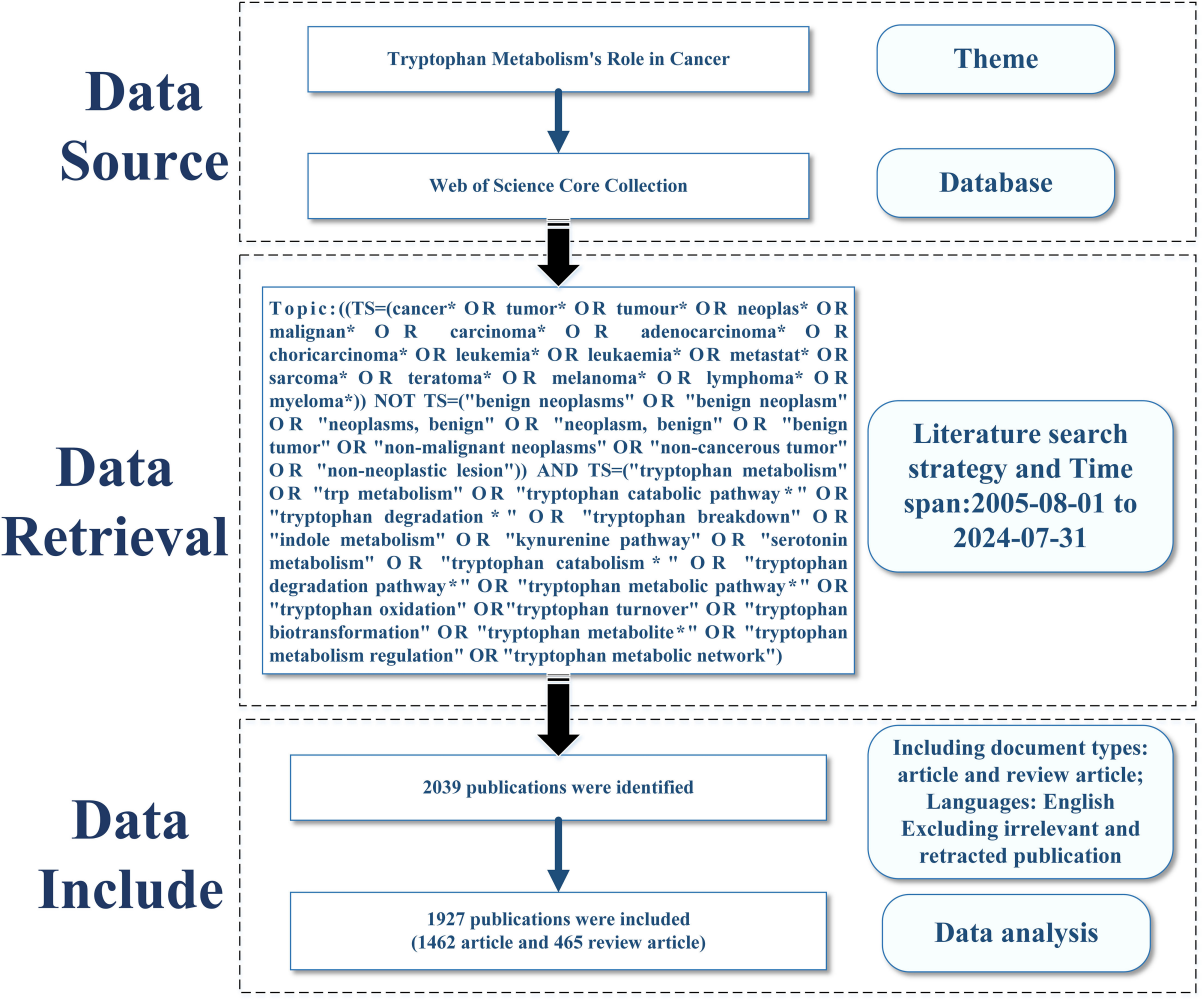
Flowchart of the literature screening process.

To ensure the scientific rigor and reliability of the analysis, this study focused on publications from August 1, 2005, to July 31, 2024. Only English-language original research articles and reviews were included, while unrelated or retracted publications were excluded. After applying these criteria, 1,927 high-quality articles were retained for analysis. All retrieved records were exported in plain text format in multiple batches, each named as “download_xxx.txt,” including full records and cited references. The data were then imported into CiteSpace software for duplicate removal, resulting in a final dataset comprising 1,927 valid articles. The entire data retrieval and preprocessing process was completed on August 1, 2024. A detailed workflow is illustrated in **Figure 2**. To enhance the comprehensiveness and transparency of this study—and to acknowledge the contributions of early researchers—**Supplementary Table S1** provides the annual publication distribution from 1955 to 2004, along with a brief summary of several representative early studies.

### 2.3 Analysis tools

This study employed a range of visualization tools to intuitively and systematically present and analyze bibliometric data, including Python, CiteSpace (6.3.R1), VOSviewer (1.6.19), and R Bibliometrix. Python was primarily used for flexible data processing and the generation of customized visualizations, such as annual publication trends and comparative metric charts. CiteSpace facilitated data cleaning and comprehensive visual analyses, including institutional collaboration networks, co-cited references, keyword co-occurrence, and clustering. VOSviewer and R Bibliometrix were applied to construct collaboration and co-occurrence networks among countries, authors, and journals, providing a clear depiction of global research collaboration patterns and the distribution of academic influence.

To systematically evaluate the research landscape and identify emerging trends in tryptophan metabolism and cancer, several core bibliometric indicators were applied. The number of publications was used to assess research activity and temporal evolution, while citation frequency reflected academic impact. Author and institutional performance were evaluated using the H-index, G-index, and M-index. Keyword co-occurrence frequency and centrality helped identify key research hotspots and core themes. Additionally, burst detection was conducted to uncover rapidly emerging topics within specific timeframes. Clustering analyses of keywords and co-cited references were also performed. The quality and robustness of the clustering structure were assessed using modularity (Q) and silhouette (S) scores. A Q value greater than 0.3 indicates statistically significant clustering. An S value above 0.5 suggests reasonable cluster quality, while a value above 0.7 reflects high reliability and efficiency.

## 3 Results

### 3.1 Analysis of publications and citations

**Figure 3:**
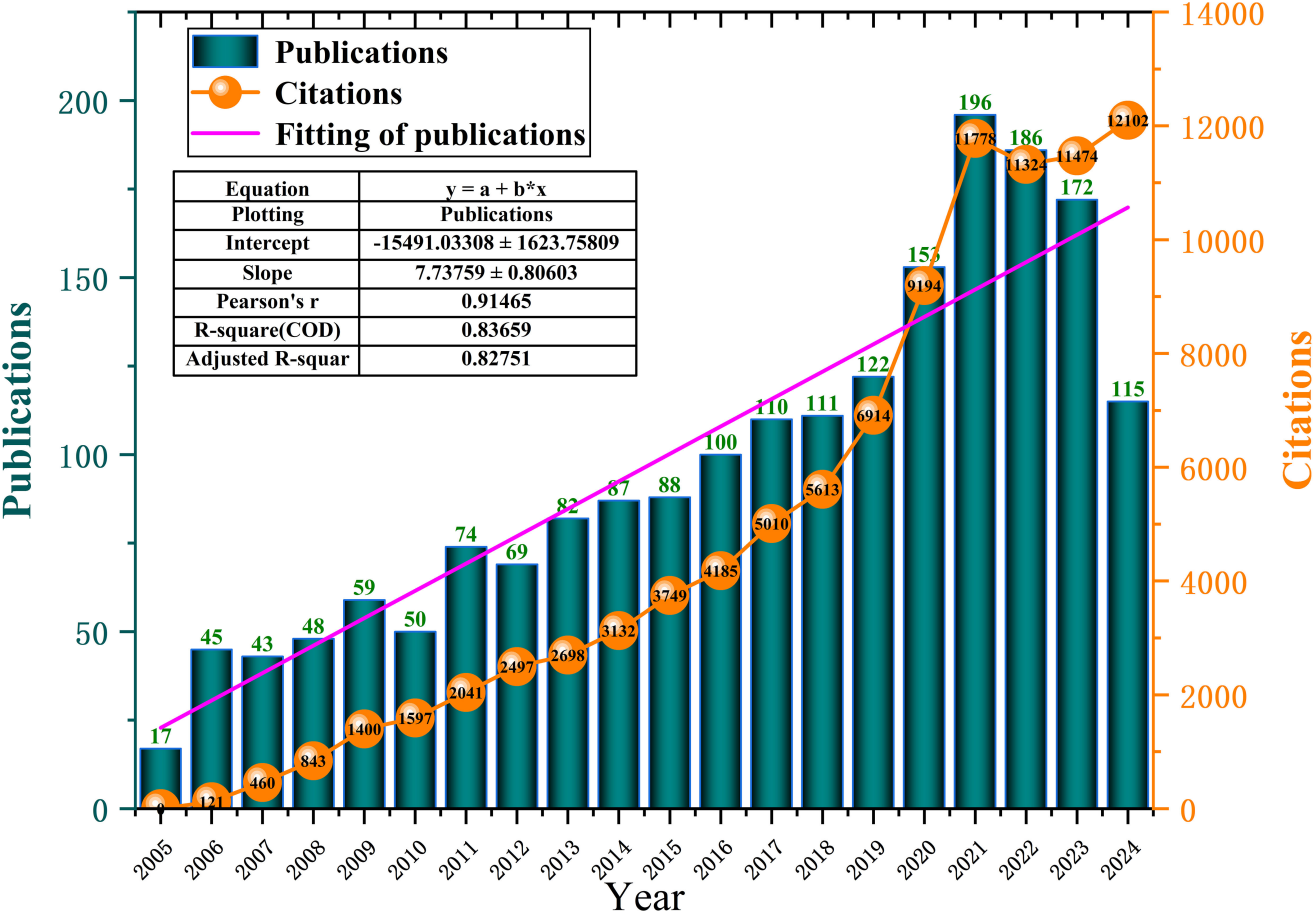
Annual publication and citations on tryptophan metabolism in cancer research from 2005 to 2024.

Between 2005 and 2024, a total of 1,927 publications related to tryptophan metabolism in cancer were identified (see **Figure 3**). The field began with just 17 publications in 2005 and has since demonstrated steady growth. Notably, research activity accelerated significantly after 2019, reaching a peak of 196 publications in 2021. Although there was a slight decline in output in 2022 and 2023, the overall upward trajectory remains strong. This temporary dip may be partly attributed to the global COVID-19 pandemic, which disrupted research activities worldwide. Simultaneously, citation counts increased dramatically—from zero in 2005 to 12,102 by 2024—indicating growing academic attention and influence. Linear regression analysis demonstrated a strong positive correlation between publication volume and year (R² = 0.83659, Adjusted R² = 0.82751), suggesting a high degree of model fit and confirming the robust upward trend. These findings highlight the increasing importance of tryptophan metabolism in cancer research and the expanding interest in this topic over the past two decades.

### 3.2 Analysis of the top producing countries/regions

A total of 1,927 publications related to tryptophan metabolism in cancer were contributed by researchers from 70 countries. As shown in **Table 1**, the United States leads in publication output with 537 papers and 40,276 citations, and demonstrates a total link strength of 320, highlighting its strong academic influence and extensive research collaborations. China ranks second with 529 publications, a total link strength of 120, and 14,522 citations. Although China’s publication count nearly matches that of the United States, a significant gap in citation frequency suggests room for improvement in research quality and international impact. Germany, despite publishing only 161 papers, has garnered 10,810 citations and achieved a total link strength of 214, reflecting its prominent role within the European research network and its high academic influence.

**Table 1:** Top 10 countries/regions and institutions contributing to tryptophan metabolism research in cancer.

Rank	Country	Publications	Citations	Total link strength	Institutions	Publications	Institutions	Citations
1	USA	537	40276	320	Medical University of Innsbruck	144	German Cancer Research Center (DKFZ)	3508
2	China	529	14522	120	Wayne State University	135	Thomas Jefferson University	3285
3	Germany	161	10810	214	Helmholtz Association	122	Lankenau Institute for Medical Research	3243
4	Italy	120	8383	130	German Cancer Research Center (DKFZ)	107	Medical College of Georgia	2959
5	United Kingdom	119	6181	205	Ruprecht Karls University Heidelberg	105	University of Perugia	2888
6	Japan	109	6121	39	University of Texas System	100	Innsbruck Medical University	2421
7	Austria	85	4451	73	University of California System	96	University of Illinois	2321
8	Australia	79	4945	114	Harvard University	89	Catholic University of Louvain	2303
9	France	79	5925	160	Northwestern University	79	University of Padua	2108
10	Netherlands	75	4987	119	Medical University of Lublin	78	University of Sydney	1986

**Figure 4:**
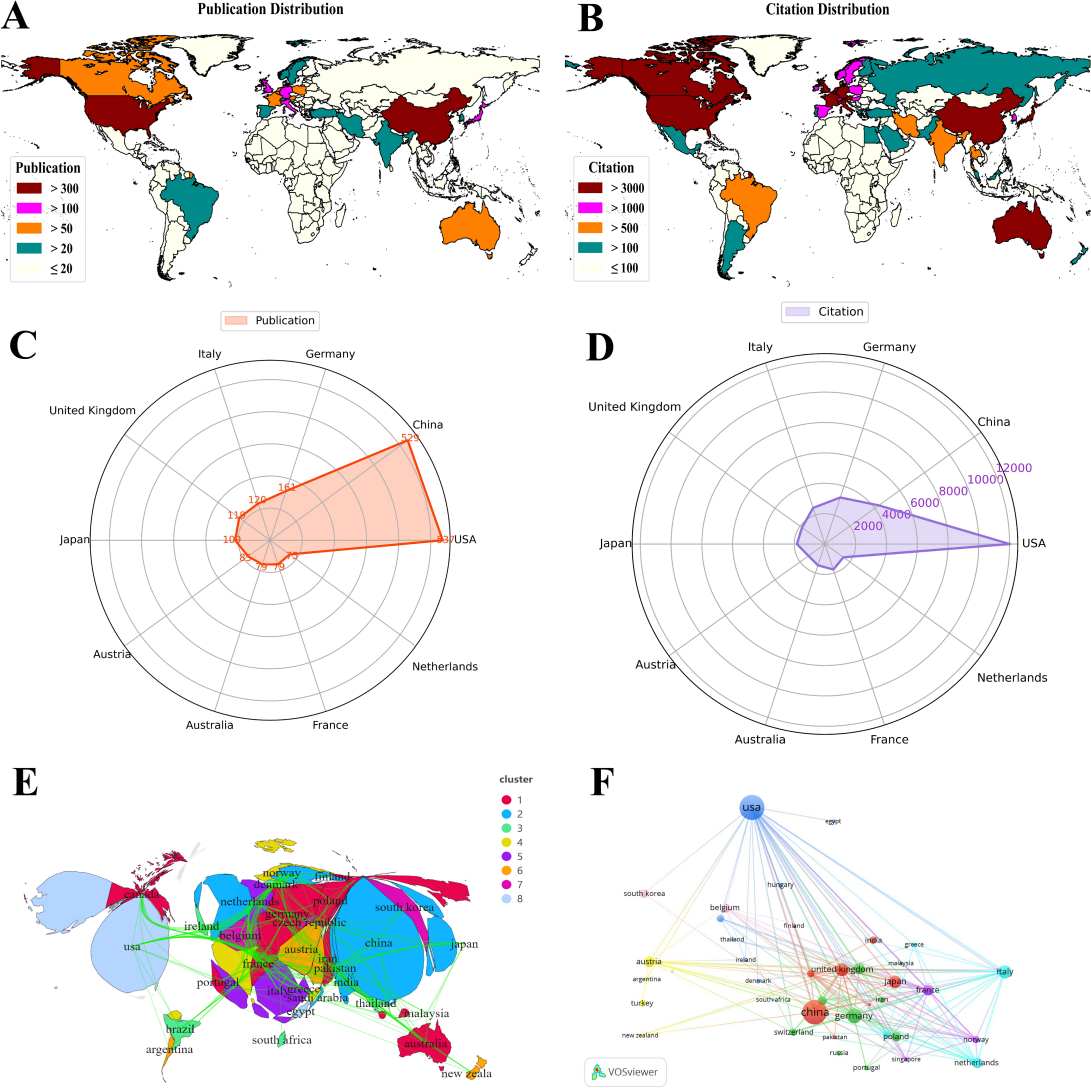
Global distribution of tryptophan metabolism in cancer research. **(A)** Global distribution of publications on tryptophan metabolism in cancer, with colors representing varying publication volumes, and darker red indicating higher counts. **(B)** Global distribution of citations on tryptophan metabolism in cancer, with colors representing varying citation frequencies, and darker red indicating higher counts. **(C)** Visualization map of country publications in tryptophan metabolism and cancer. **(D)** Visualization map of country citations in tryptophan metabolism and cancer. **(E)** Geographic clustering visualization of countries/regions. **(F)** Collaboration network diagram of countries/regions in tryptophan metabolism and cancer, where nodes represent countries, node size reflects publication volume, and links indicate collaboration strength.

The global distribution of research efforts (see **Figures 4A, B**) shows that the United States and China dominate in both publication volume and citation frequency. In contrast, European countries such as Germany, the United Kingdom, and France exhibit a clear advantage in research quality and international collaboration. Radar charts (see **Figures 4C, D**) further illustrate the comparative academic output and collaborative strengths of leading countries. Cluster analysis (**Figure 4E**) reveals the formation of several regional and international research alliances, underscoring a pattern of strong transnational collaboration. The United States plays a central role in the global research network, engaging extensively with partners across Europe, Asia, and beyond. In comparison, countries in South America, Africa, and parts of Southeast Asia remain underrepresented, with their research activities often relying on collaborations with major contributors such as the United States and China. To further visualize national contributions, 41 countries with at least five publications were selected for network mapping (see **Figure 4F**). The resulting distribution confirms clear regional and collaborative patterns, highlighting both the global scope and the geographic disparities in research on tryptophan metabolism and cancer.

### 3.3 Analysis of the top-producing authors

A total of 11,134 authors worldwide have contributed to 1,927 publications in the field of tryptophan metabolism and cancer. The top ten authors, ranked by the number of publications (NP), were further evaluated based on key bibliometric indicators, including citation frequency, H-index, G-index, and M-index (see **Table 2**). The H-index measures a researcher’s sustained impact by quantifying the number of publications (h) that have been cited at least h times. The G-index builds on this by giving additional weight to highly cited papers, thus reflecting the breadth of a scholar’s influence. The M-index, calculated as H/N (where N is the number of years since the researcher’s first publication), captures the pace of academic impact over time, with higher values indicating a more rapid trajectory of influence (26).

**Table 2:** Top 10 authors contributing to tryptophan metabolism research in cancer.

Rank	Author	NP	TC	h_index	g_index	m_index	PY_start
1	Fuchs, Dietmar	61	3560	29	59	1.526	2006
2	Prendergast, George C	25	3628	23	25	1.15	2005
3	Mittal, Sandeep	22	631	18	22	1.125	2009
4	Takikawa, Osamu	22	1934	18	22	0.9	2005
5	Guillemin, Gilles J	22	1775	16	22	0.889	2007
6	Wang Y	22	861	11	22	0.786	2011
7	Platten, Michael	21	3178	19	21	1.056	2007
8	Saito, Kuniaki	21	785	18	21	0.947	2006
9	Juhász, Csaba	21	635	17	21	0.895	2006
10	Muller, Alexander J	19	2762	18	19	0.9	2005

**Figure 5:**
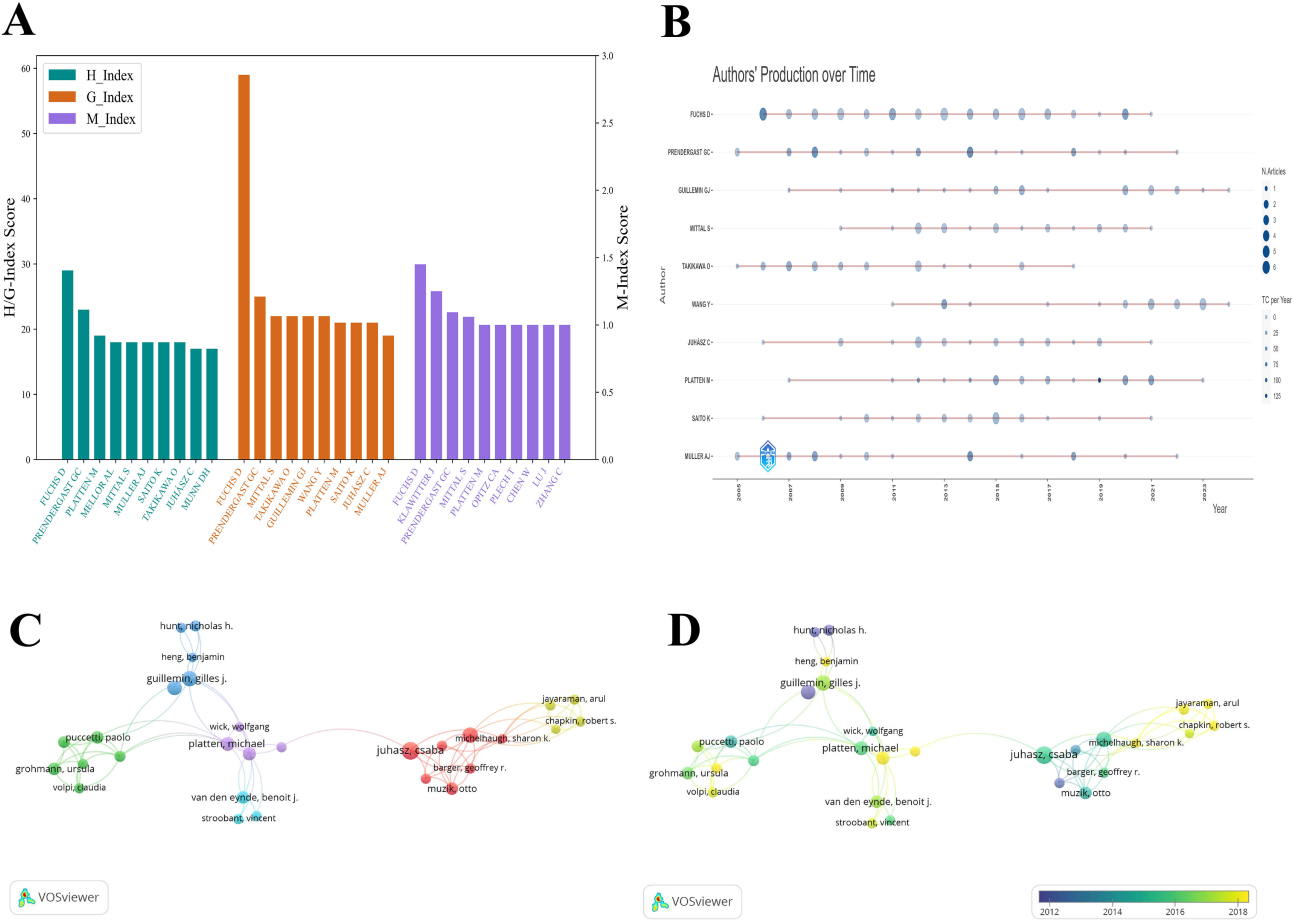
Visualization of the top authors in tryptophan metabolism research in cancer. **(A)** Visualization map of H-index, G-index, and M-index for the top 10 authors by publication volume, with colors representing different impact metrics. **(B)** Publication volume of the top 10 authors over time. Node size represents the number of articles, and color intensity reflects total citations. **(C)** Author co-citation network map in tryptophan metabolism and cancer, with colors representing distinct collaboration groups. **(D)** Temporal trend graph of the author co-citation network in tryptophan metabolism and cancer, with color intensity ranging from purple to yellow to represent the progression of years from past to present.

Among the top contributors, Fuchs, Dietmar ranks first with 61 publications and holds the highest H-index (29), G-index (59), and M-index (1.526), reflecting both prolific output and sustained scholarly influence. Although Prendergast, George C has authored fewer papers (25), he has received 3,628 citations—surpassing Fuchs—and his M-index of 1.15 suggests a fast-growing academic presence. Mittal, Sandeep, Takikawa, Osamu, and Guillemin, Gilles J show comparable H-indices (18, 18, and 16, respectively) and G-indices (all 22), indicating similar levels of research contribution. However, Mittal’s M-index (1.125) points to a faster growth rate in scholarly influence compared to Takikawa (0.9) and Guillemin (0.889). Although Müller, Alexander J has the fewest publications (19), his H-index (18) and M-index (0.9) indicate a relatively high quality of work with steady, albeit slower, impact growth. Overall, Fuchs, Prendergast, and Mittal stand out as key figures in the field, each demonstrating unique patterns of academic influence (see **Figures 5A, B**).

Regarding collaboration networks, Juhasz, Csaba and Mittal, Sandeep play central roles, with total link strengths of 100 and 99, respectively, underscoring their importance in facilitating scientific collaboration. Co-authorship network visualizations (**Figures 5C, D**) further illustrate these relationships. In **Figure 5C**, clusters are color-coded by research themes, node size represents publication volume, and line thickness indicates the strength of collaboration. The red cluster, containing the largest number of authors, represents a robust collaborative group, while the purple cluster shows strong connections to other clusters, highlighting cross-disciplinary interactions. **Figure 5D** adds a temporal dimension, showing that Juhasz was most actively involved in collaborative work between 2016 and 2018. In recent years, researchers such as Jayaraman, Arul, Chapkin, Robert S, and Opitz, Christiane A have emerged as active participants in evolving collaboration networks.

### 3.4 Analysis of the top-producing institutions

**Figure 6:**
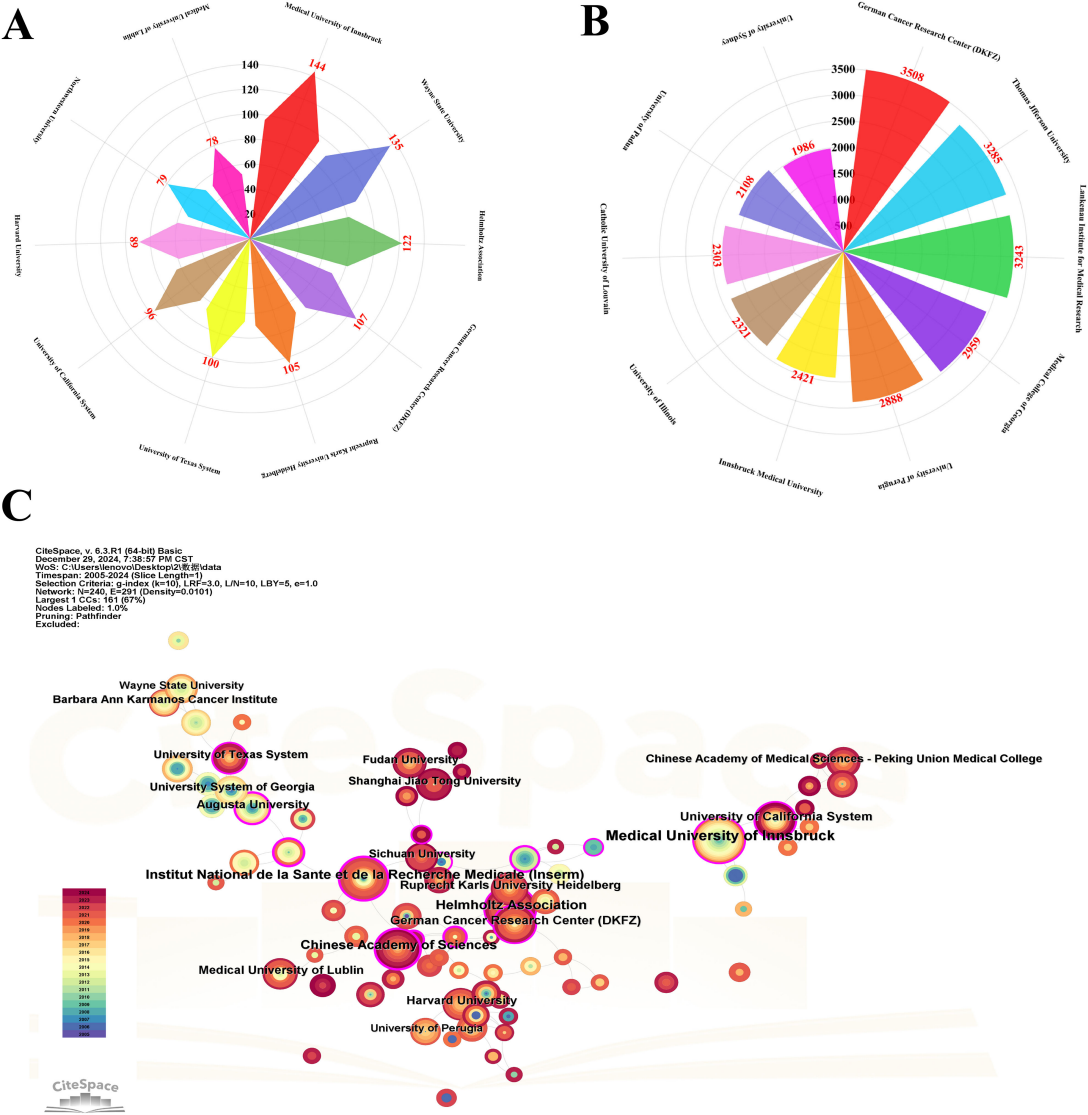
Visualization of the top institutions in tryptophan metabolism research in cancer. **(A)** Visualization map of the top 10 institutions based on publication volume. **(B)** Visualization map of the top 10 institutions based on citation count. **(C)** Collaborative relationship map among research institutions. Nodes represent individual institutions, with node size corresponding to publication volume. The thickness of connecting lines reflects collaboration strength, and the purple outer ring around each node indicates betweenness centrality.

In the visualization analysis of institutional output, influence, and collaboration in tryptophan metabolism and cancer research, **Figures 6A, B** display the top ten institutions ranked by publication volume and citation frequency, respectively. Publication volume—a direct measure of institutional output—shows that the Medical University of Innsbruck leads the field with 144 publications, indicating its high level of research activity. It is followed by Wayne State University (135 publications) and the Helmholtz Association (122 publications). The German Cancer Research Center (DKFZ) ranks fourth with 107 publications, reflecting its sustained contributions to the field (see **Table 1**). **Figure 6B**, which highlights citation frequency as a measure of academic impact, shows that DKFZ leads with 3,508 citations, underscoring both the volume and influence of its research. Thomas Jefferson University and the Lankenau Institute for Medical Research follow with 3,285 and 3,243 citations, respectively, further emphasizing their significant academic standing in this domain. A comparison of **Figures 6A, B** reveals that academic institutions dominate both in output and impact, likely due to their central roles in conducting foundational and innovative research. These data provide valuable insights into the current institutional landscape and offer direction for future research efforts and collaborative partnerships.

**Figure 6C** visualizes the collaborative network among institutions from 2005 to 2024. Each node represents a research institution, with node size proportional to its publication volume, and line thickness indicating the strength of collaboration. The Medical University of Innsbruck appears as the largest node, reflecting its dominant role in the field, followed by the Helmholtz Association, Institut National de la Santé et de la Recherche Médicale (Inserm), the Chinese Academy of Sciences, and DKFZ. The purple outer ring around each node represents betweenness centrality, which reflects an institution’s role as a connector within the collaborative network. A total of eight institutions have a betweenness centrality ≥ 0.2, highlighting their key positions in facilitating inter-institutional cooperation. Gustave Roussy exhibits the highest betweenness centrality (0.43), underscoring its pivotal bridging role. Both the Chinese Academy of Sciences and the National Center for Geriatrics & Gerontology show values of 0.28, further indicating their strategic importance within the global collaboration network.

### 3.5 Analysis of the top-producing journals

**Figure 7:**
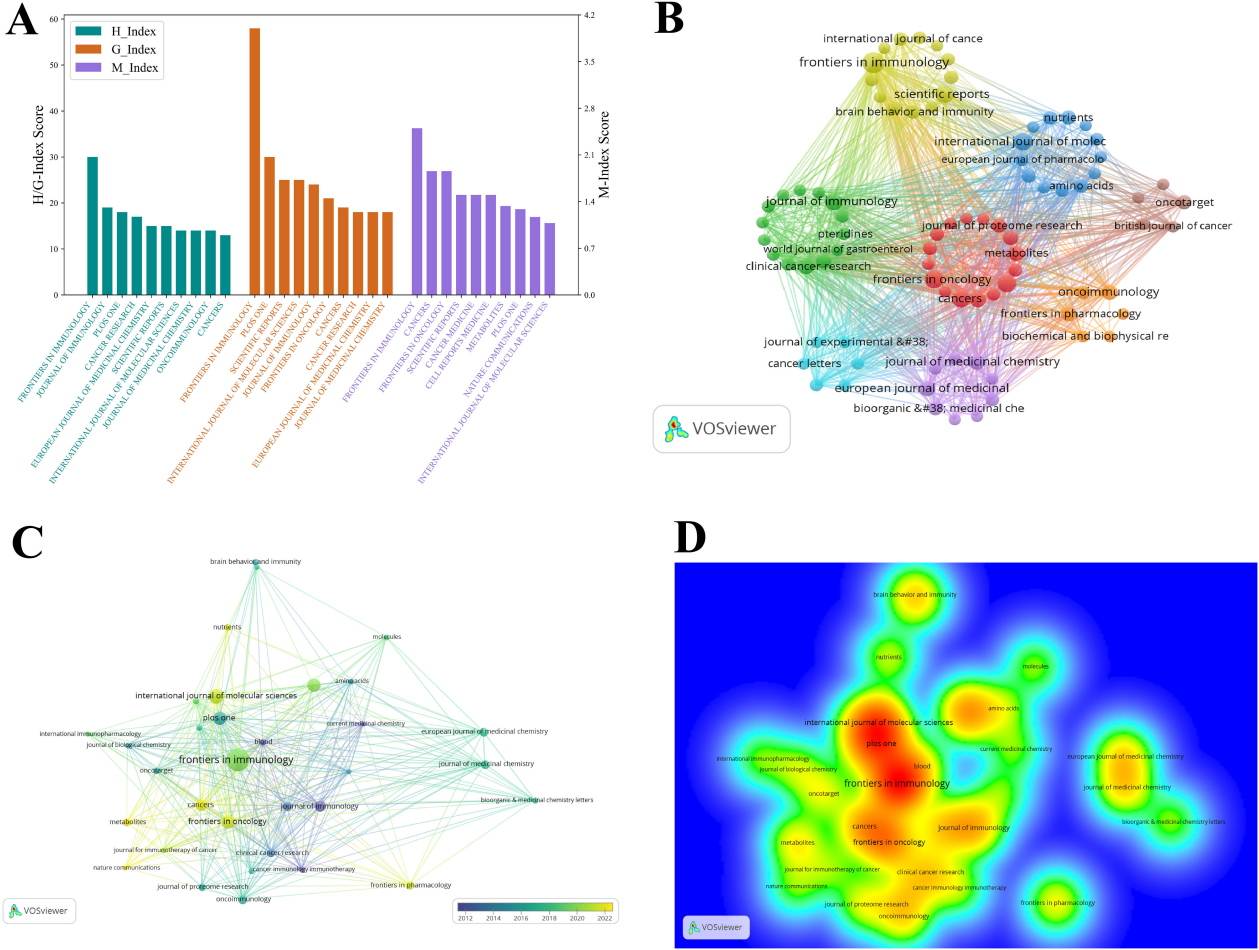
Visualization of the top journals in tryptophan metabolism research in cancer. **(A)** Visualization map of H-index, G-index, and M-index for the top 10 journals by publication volume, with colors representing different impact metrics. **(B)** Journal thematic clustering map of tryptophan metabolism and cancer research. Nodes represent distinct journal clusters, with colors denoting different research themes. **(C)** Temporal trend graph of the journal collaboration network in tryptophan metabolism and cancer, with color intensity shifting towards yellow indicating increased collaboration in recent years. **(D)** The item density map visualizes the distribution density of journals within the collaboration network, with color intensity reflecting citation frequency and collaboration strength.

The academic impact of a journal serves as a key indicator of its standing within the scientific community. By examining metrics such as the H-index, G-index, and M-index, a more comprehensive evaluation of a journal’s citation performance and scholarly value can be obtained. In the field of tryptophan metabolism and cancer, a total of 781 journals have contributed to the dissemination of research, playing a critical role in advancing knowledge and academic communication. To gain deeper insights into the publication patterns within this field, we conducted a focused analysis of the top ten journals by publication volume (see **Table 3**). Among them, *Frontiers in Immunology* demonstrated outstanding performance across multiple metrics, including publication volume, citation frequency, H-index, G-index, and M-index, highlighting its significant academic influence in the field of immunology (see **Figure 7A**). The *International Journal of Molecular Sciences* and *Plos One* ranked second and third in terms of publication volume, respectively. However, *Plos One* recorded a substantially higher citation count. Although *Frontiers in Oncology* published a comparable number of papers to *Plos One*, its total citations remained lower, indicating a relatively lesser impact. Notably, several journals—such as *Journal of Immunology*, *Cancer Research*, and *Journal of Medicinal Chemistry* — published fewer articles but achieved high citation frequencies, reflecting the strong academic recognition of their contributions. Furthermore, eight out of the ten leading journals are classified as Q1 journals, underscoring the high visibility and scholarly esteem that research on tryptophan metabolism and cancer has garnered within the scientific community.

**Table 3:** Top 10 journals contributing to tryptophan metabolism research in cancer.

Rank	Source	NP	TC	h_index	g_index	m_index	IF	JCR
1	*Frontiers in Immunology*	61	3379	30	58	2.308	5.7	Q1
2	*International Journal of Molecular Sciences*	34	656	14	25	1	4.9	Q1
3	*Plos One*	30	1107	18	30	1.2	2.9	Q1
4	*Frontiers in Oncology*	30	496	13	21	1.625	3.5	Q2
5	*Scientific Reports*	29	646	15	25	1.364	3.8	Q1
6	*Cancers*	25	391	13	19	1.625	4.5	Q1
7	*Journal of Immunology*	24	1671	19	24	0.95	3.6	Q2
8	*Cancer Research*	18	2535	17	18	0.81	12.5	Q1
9	*European Journal of Medicinal Chemistry*	18	512	15	18	0.938	6.0	Q1
10	*Journal of Medicinal Chemistry*	18	1223	14	18	0.778	6.8	Q1

The journal thematic clustering map (see **Figure 7B**) offers a clear visualization of the thematic distribution of journals within the field of tryptophan metabolism and cancer, revealing their affiliations based on subject similarity. In the figure, nodes of different colors represent distinct clusters, each corresponding to collaborative networks centered around specific research themes. Overall, the clusters form interconnected structures, highlighting the interdisciplinary nature of this research area. The yellow cluster primarily focuses on immunology, with core journals including *Frontiers in Immunology*, *Scientific Reports*, *Brain*, *Behavior*, and *Immunity*, and *International Journal of Cancer*. Among these, *Frontiers in Immunology* emerges as the most influential node, distinguished by its high publication volume, citation frequency, and academic impact. This highlights the central role of immunological mechanisms—particularly immunometabolic regulation and immunotherapy—in research on tryptophan metabolism and cancer. The red cluster is predominantly associated with cancer research, encompassing themes such as cancer metabolism, proteomics, and clinical treatment strategies. Representative journals within this cluster include *Frontiers in Oncology*, *Cancers*, *Journal of Proteome Research*, and *Metabolites*. The high thematic coherence and strong inter-journal connections observed in this cluster underscore the critical role of tryptophan metabolism in cancer initiation, progression, and therapeutic development.

The blue cluster is primarily associated with molecular biology and pharmacology research. Key journals in this cluster include the *European Journal of Pharmacology*, *Amino Acids*, and *Nutrients*. The presence of this cluster underscores the significance of tryptophan metabolism not only in cancer pathophysiology but also in areas such as molecular signaling, nutritional metabolism, and drug development. Research within this domain typically adopts a multidisciplinary approach, integrating biochemistry, bioinformatics, and pharmacology, and thereby offering innovative strategies and insights for cancer therapy. The green cluster focuses predominantly on clinical applications, particularly in the fields of immunology and cancer treatment. Representative journals include *Journal of Immunology* and *Clinical Cancer Research*. The formation of this cluster reflects the increasing convergence of basic research and clinical translation, with discoveries in tryptophan metabolism being progressively applied to precision medicine and the development of personalized therapeutic strategies. In recent years, growing attention has been directed toward the role of immunometabolic regulation in cancer therapy, and the existence of this cluster further substantiates this emerging trend.

To further investigate the evolution of journals in this field, we analyzed temporal trends to reveal changes in academic influence and shifting research hotspots (see **Figure 7C**). Between 2018 and 2022, journals such as *Frontiers in Immunology*, *Frontiers in Pharmacology*, *Frontiers in Oncology*, and *International Journal of Molecular Sciences* have shown increased activity within the collaboration network, indicating their rising prominence in tryptophan metabolism and cancer research. Conversely, some established journals, including the *Journal of Immunology*, despite maintaining high academic influence, are gradually losing centrality in the network to these emerging outlets. This transition reflects a shift in research focus from traditional immune mechanism studies toward more interdisciplinary directions, particularly at the intersection of immunotherapy and cancer metabolism. The density map (see **Figure 7D**) further illustrates the spatial distribution of collaboration hotspots and centers of influence among journals. High-density regions (red) cluster around *Frontiers in Immunology*, *Plos One*, and *International Journal of Molecular Sciences*, underscoring their pivotal roles in citation frequency and collaborative engagement, thereby forming the core of the field’s influence. Medium-density areas (yellow) include journals such as *Frontiers in Oncology*, *Cancers*, and *Journal of Immunology*, which exert notable influence within specific subdomains but have yet to integrate fully into the core collaborative network. Journals situated in low-density regions (blue) occupy the periphery, often representing niche or emerging topics, thus reflecting unique research trajectories and collaboration patterns within the broader field.

### 3.6 Analysis of the top-cited references

Local Citations (LC) and Global Citations (GC) are critical metrics for assessing the scholarly impact of individual publications. LC measures a paper’s influence within a specific research domain—such as tryptophan metabolism in cancer—while GC captures its overall recognition across the broader scientific community. The LC/GC ratio (local-to-global citation ratio) provides further insight into the paper’s relative impact: a higher ratio indicates strong influence within a specialized field, but potentially limited reach beyond it. As shown in **Table 4**, Platten M (2019, Nat Rev Drug Discov) received 166 local citations and 897 global citations, highlighting its substantial academic impact both within the field and across related disciplines, such as drug development and immunotherapy. In contrast, Pilotte L (2012, P Natl Acad Sci USA) accumulated 192 Local Citations but only 471 Global Citations, suggesting concentrated recognition within tryptophan metabolism and cancer research, yet more limited dissemination in the wider scientific landscape.

**Table 4:** Top 10 most cited references on tryptophan metabolism in cancer.

Rank	Document	DOI	Year	Local citations	Global citations	LC/GC ratio (%)	Normalized local citations	Normalized global citations
1	CLIN CANCER RES (27)	10.1158/1078-0432.CCR-05-1966	2006	206	522	39.46	11.21	4.68
2	P NATL ACAD SCI USA (28)	10.1073/pnas.1113873109	2012	192	471	40.76	12.96	6.1
3	J CLIN INVEST (29)	10.1172/JCI31178	2007	185	872	21.22	6.99	6.14
4	PLATTEN M, 2019, NAT REV DRUG DISCOV (30)	10.1038/s41573-019-0016-5	2019	166	897	18.51	29.06	20.25
5	CANCER RES (31)	10.1158/0008-5472.CAN-07-1872	2007	163	408	39.95	6.16	2.87
6	CANCER RES (32)	10.1158/0008-5472.CAN-12-0569	2012	156	539	28.94	10.53	6.98
7	BLOOD (33)	10.1182/blood-2009-09-246124	2010	122	440	27.73	8.28	5.37
8	CANCER IMMUNOL IMMUN (34)	10.1007/s00262-008-0513-6	2009	109	260	41.92	7.13	2.53
9	J CLIN INVEST (35)	10.1172/JCI31911	2007	107	665	16.09	4.04	4.68
10	NAT REV CANCER (36)	10.1038/nrc2639	2009	103	360	28.61	6.74	3.5

**Figure 8:**
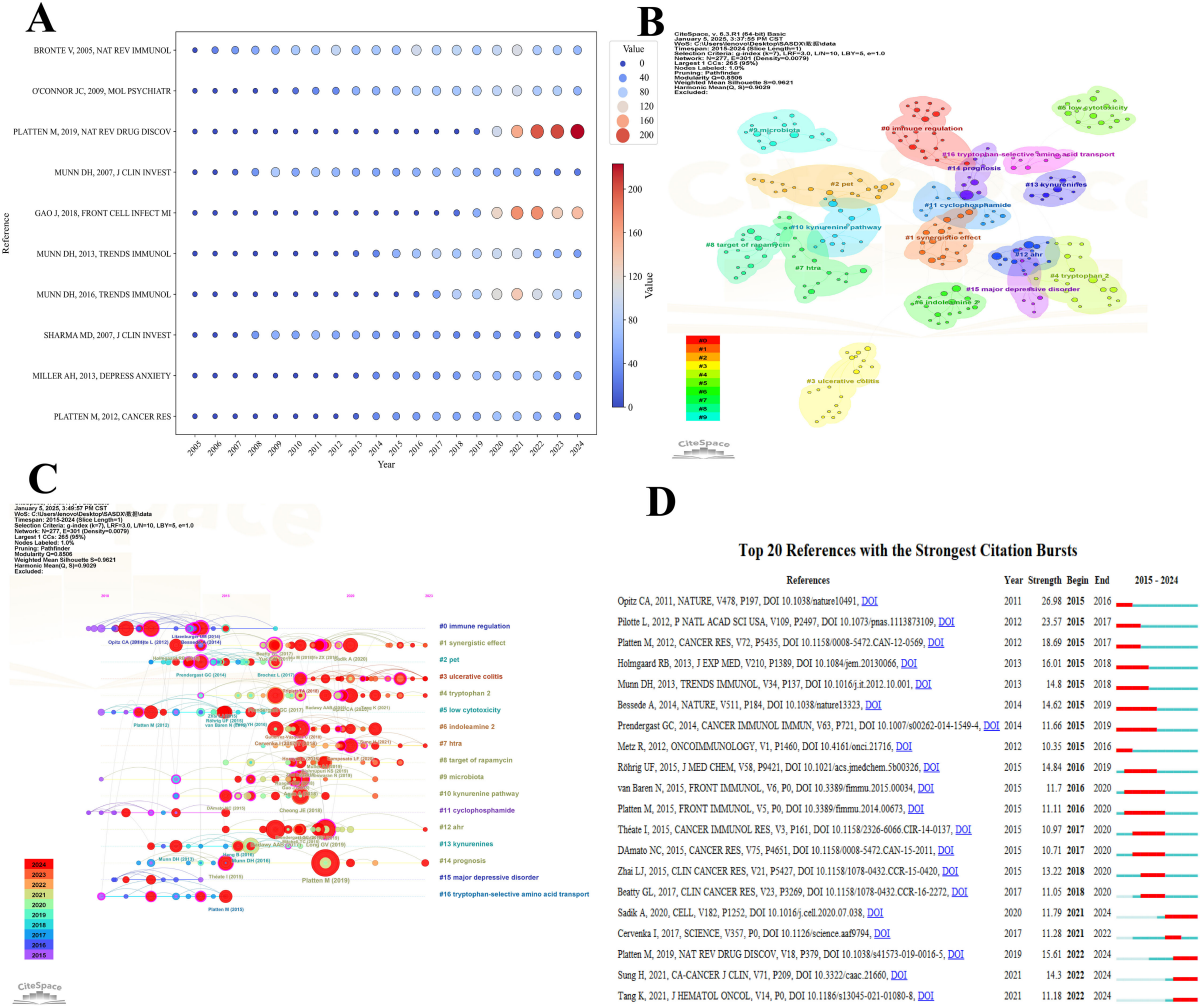
Visualization of the top cited references in tryptophan metabolism research in cancer. **(A)** Visualization map of the top 10 most cited references over the past 20 years, illustrating the temporal variation in citation frequency. Each bubble represents a publication, with its position corresponding to the citation count in a specific year. The size and color intensity of the bubbles reflect the citation frequency of the publication. **(B)** Thematic clustering map of the cited literature, where node size reflects citation frequency and node color represents distinct research themes. **(C)** The top sixteen clusters timeline distribution. Each node signifies a publication, with its size corresponding to the citation frequency in its respective year. The node colors transition from blue (2015) to red (2023), representing the temporal progression. Lines connect publications within the same cluster across various years, showcasing the temporal continuity and evolutionary paths of research within each cluster. **(D)** The top 20 references with the highest citation burst. A red bar represents a significant increase in citations during the corresponding year.

Citation frequency is a key indicator of a publication’s academic impact, often reflecting its contribution to theoretical innovation, methodological advancement, or technological application. Highly cited studies frequently serve as foundational or landmark references, guiding subsequent research in the field. As illustrated in **Figure 8A**, each bubble represents a publication, with its position corresponding to the number of citations received in a given year. The size and color of the bubbles indicate citation magnitude—larger, red bubbles denote high citation counts, while smaller, blue ones reflect lower frequencies. Platten M (2019, Nat Rev Drug Discov) has shown a significant rise in citations since 2019, reaching 223 citations by 2024, making it one of the most highly cited publications in the dataset. Similarly, Gao J (2018, Front Cell Infect Microbiol) experienced a rapid increase in citations from 2020 to 2024, reflecting its growing academic attention in later years. In contrast, earlier publications such as Bronte V (2005, Nat Rev Immunol) maintained high citation levels from 2010 to 2021 but have since shown a gradual decline, suggesting a waning influence over time. **Figure 8B** presents a thematic clustering map of the cited literature, identifying 16 distinct research themes. Each colored region represents a thematic cluster, with node size indicating citation frequency and color denoting thematic category. Cluster #0, labeled “immune regulation,” is the largest, suggesting it is the most extensively studied area and likely represents a central research axis in the field. The partial overlap among clusters underscores the interdisciplinary nature of tryptophan metabolism research, highlighting the close integration of immunology, oncology, metabolism, and therapeutic development.

**Figure 8C** presents a timeline visualization of citation trends and the evolution of research themes in the field. Red circles denote highly cited publications, with their size proportional to citation frequency, while connecting lines illustrate thematic or citation linkages between studies. This timeline effectively captures the emergence, development, and transition of key research foci over time. Between 2015 and 2019, themes such as #0 immune regulation and #10 kynurenine pathway experienced rapid growth, with associated publications receiving substantial academic attention. Notably, since 2019, newer themes—such as #15 major depressive disorder and #3 ulcerative colitis—have shown a significant increase in citation frequency, reflecting a shift in research interest toward the broader implications of tryptophan metabolism in neuroimmunological and inflammatory disorders. Key studies, including Platten M (2019) and Opitz CA (2011), occupy pivotal positions on the timeline, suggesting their role as landmark contributions that have shaped the trajectory of the field. Collectively, the visualization underscores not only the dynamic nature of research priorities but also the expanding interdisciplinary relevance of tryptophan metabolism in cancer and related pathologies.

**Figure 8D** highlights the top 20 publications with the highest burst strength, reflecting research that experienced a rapid and concentrated increase in scholarly attention over specific time intervals. Red bars indicate the duration of each citation burst, illustrating both the timing and intensity of influence. Most citation bursts occurred between 2015 and 2020, suggesting that this timeframe marked a critical phase in the maturation of the field. The majority of these burst-identified publications focus on key themes such as immune regulation, metabolic pathways, and clinical therapeutics, emphasizing their centrality in shaping research directions. Among them, Platten M (2019) stands out with a burst strength of 26.18, beginning in 2019 and continuing through 2024, signifying its profound and sustained academic impact. In contrast, Opitz CA (2011), though published earlier, experienced its burst primarily during 2015–2016, indicating a delayed but notable rise in recognition. Similarly, Munn DH (2013) and Bessede A (2014) showed pronounced bursts from 2015 to 2018, likely corresponding to inflection points in the development of immunometabolic and therapeutic research within the field.

### 3.7 Analysis of the top keywords

**Figure 9:**
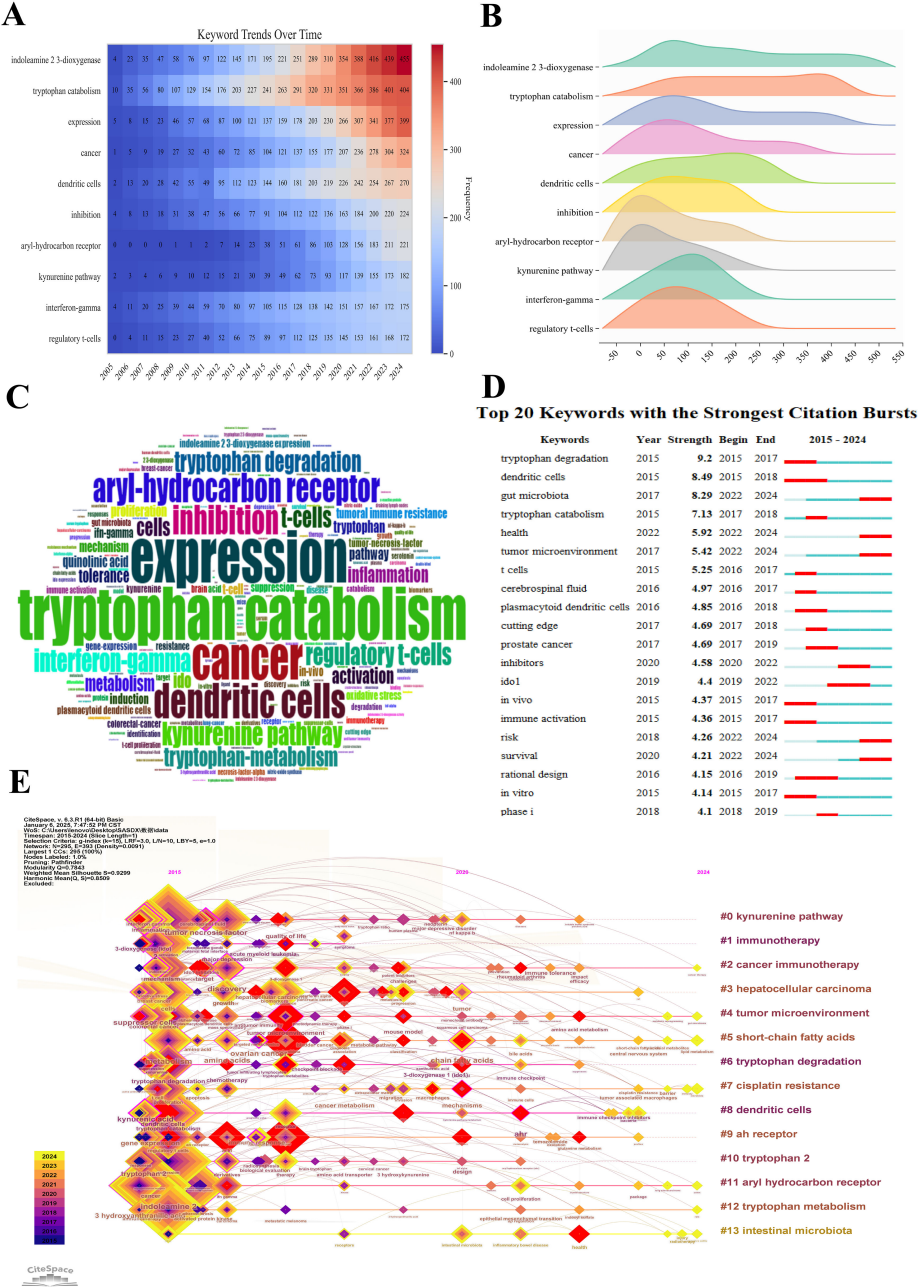
Visualization of the top keywords in tryptophan metabolism research in cancer. **(A)** Heatmap of keyword distribution over time in tryptophan metabolism and cancer research. The redder the color, the higher the co-occurrence frequency of keywords. **(B)** Ridge plot depicting the distribution patterns of the top 10 most frequently co-occurring keywords in tryptophan metabolism and cancer research. The length of each color distribution indicates a higher frequency of occurrence within that range, signifying the sustained attention these keywords have received in the literature. **(C)** Word cloud of the top 200 keywords in tryptophan metabolism and cancer research. The size of the keywords is proportional to their co-occurrence frequency. **(D)** The top 20 keywords with the strongest citation bursts. A red bar represents a significant increase in citations during the corresponding year. **(E)** Timeline visualization of keyword evolution based on CiteSpace. Node size reflects the research impact of keywords, with larger nodes indicating greater influence. Colors shift from purple (earlier years) to yellow (later years), showing time progression. Lines show connections between keywords, highlighting research topic relationships and development trends.

By analyzing the keywords in the field of tryptophan metabolism and cancer, we can delve into the research hotspots and future trends within this domain. The heatmap in **Figure 9A** provides a clear illustration of the temporal trends in keyword co-occurrence frequency. The gradient from blue to red intuitively reflects the transition from lower to higher frequency values. Notably, the keywords “indoleamine 2,3-dioxygenase” and “tryptophan catabolism” have shown a consistent upward trend, underscoring their central role in the expanding intersection of immunology and cancer biology. Similarly, the increasing prevalence of terms such as “cancer” and “dendritic cells” reflects the rapid evolution of cancer immunotherapy and dendritic cell-based research. In recent years, keywords like “aryl-hydrocarbon receptor” and “kynurenine pathway” have exhibited accelerated growth, suggesting heightened interest in their roles as emerging regulators of immune response and potential therapeutic targets. In contrast, foundational terms such as “expression” and “inhibition” have maintained relatively stable frequency, indicating their enduring relevance across various mechanistic studies. Additionally, the sustained rise in keywords such as “interferon-gamma” and “regulatory T cells” highlights their continued importance in immune-related research. The recurring prominence of “tryptophan catabolism”, “kynurenine pathway”, and “aryl-hydrocarbon receptor” further suggests a strong current focus on metabolic reprogramming and immunoregulatory mechanisms. Importantly, the heatmap reveals a marked surge in keyword activity between 2013 and 2015, coinciding with major advances in cancer immunology—particularly the clinical emergence of immune checkpoint inhibitors and other immunomodulatory therapies. These breakthroughs likely catalyzed the intensified research interest observed during this pivotal period.

The ridge plot presented in **Figure 9B** illustrates the distribution characteristics of the top 10 most frequently co-occurring keywords in the field. Among these, “indoleamine 2,3-dioxygenase” exhibits a distribution range of approximately between 50 and 550, indicating a high and relatively uniform occurrence frequency across this interval. This pattern suggests sustained research interest in this topic throughout the examined timeframe. Similarly, “tryptophan catabolism” displays a broad distribution from around 0 to 500, with particularly high frequencies between 300 and 400, underscoring its prominent role in the literature. In contrast, the keyword “expression” demonstrates a narrower distribution, ranging from approximately 50 to 400. Although its span is more limited, it still reflects consistent and substantial scholarly attention. Meanwhile, keywords such as “aryl-hydrocarbon receptor” and “kynurenine pathway” are primarily concentrated within the 0 to 50 range, suggesting that these topics were not focal points in the early stages of research but may have gained prominence more recently. **Figure 9C** lists the top 200 co-occurring keywords, showing a positive correlation between word frequency and occurrence rate, which reinforces the trends identified in the ridge plot. Collectively, these findings indicate that research on tryptophan metabolism and its associated immunological mechanisms has emerged as a major hotspot in recent years, reflecting the growing interest in metabolic-immunological interplay in cancer biology.

**Figure 9D** highlights the top 20 keywords with the highest burst strength in the field of tryptophan metabolism and cancer. Between 2015 and 2017, “tryptophan degradation” exhibited the strongest citation burst (strength = 9.2), ranking first among all keywords. This was followed by “dendritic cells” (8.49) and “gut microbiota” (8.29), which ranked second and third, respectively. Notably, from 2022 to 2024, keywords such as “gut microbiota”, “health”, “tumor microenvironment”, “risk”, and “survival” remained highly active, indicating a sustained and growing interest in the interplay between tryptophan metabolism, immune regulation, and the tumor microenvironment. Additionally, keywords like “inhibitors” and “IDO1” experienced marked increases in burst strength between 2019 and 2022, a trend likely driven by the rapid advancements in tumor immunotherapy. Although other keywords—such as “plasmacytoid dendritic cells”, “prostate cancer”, “*in vivo*”, “immune activation”, “rational design”, “*in vitro*”, and “phase I”—exhibited comparatively lower burst intensities, they still demonstrated notable research activity during different time periods. These keywords collectively span a wide range of research topics, from basic biological mechanisms to clinical translational applications, reflecting the depth and multidimensional nature of the field. **Figure 9E** depicts the temporal evolution of keyword clusters, categorizing research themes into 13 groups. Each node represents a specific theme, with node size corresponding to its frequency in a given year. The color gradient—from purple (2011) to yellow (2024)—visually traces the chronological development of these themes. Among them, the “kynurenine pathway” cluster stands out as a dominant and sustained hotspot, as indicated by the large node size and continued presence over time. The connecting lines between nodes illustrate co-occurrence frequencies, providing insights into the interrelationships among various research domains and emphasizing the integrative, interdisciplinary nature of this evolving field.

## 4 Discussion

### 4.1 General information

In the field of tryptophan metabolism and cancer research, a total of 1,927 papers have been published across 781 academic journals by 11,134 researchers from 70 countries worldwide. Overall, both the number of publications and citation frequency have shown a steady upward trend over the years. However, this growth has decelerated in the past two years, likely due to the global disruptions caused by the COVID-19 pandemic. The United States leads the field in terms of research output, consistently maintaining a dominant position. Among individual researchers, Dietmar Fuchs ranks first in publication count, while the Medical University of Innsbruck stands out for its sustained productivity in this domain. The German Cancer Research Center has garnered significant attention due to the high citation frequency of its publications. *Frontiers in Immunology* has emerged as a key publication platform in this domain. Notably, the review by Platten et al. (2019), published in *Nature Reviews Drug Discovery*, is among the most highly cited works in the field. This seminal paper systematically assessed the therapeutic potential of key enzymes—IDO1, IDO2, TDO, and KMO (Kynurenine 3-monooxygenase)—highlighting their roles in immune modulation and tumor immune evasion. By offering a comprehensive framework for understanding the immunological functions of tryptophan metabolism, the study has laid the groundwork for translational advances in cancer therapy.

Co-citation analyses indicate that current research on tryptophan metabolism primarily focuses on several major solid tumors, including glioblastoma, breast cancer, lung cancer, colorectal cancer, and melanoma. In glioblastoma, studies have largely concentrated on IDO1-mediated immunosuppression and its pivotal role in resistance to immune checkpoint inhibitors. Research on breast and lung cancers has highlighted the kynurenine–aryl hydrocarbon receptor (AhR) axis and serotonin signaling as critical pathways contributing to tumor progression and immune evasion. In colorectal cancer, increasing attention has been paid to gut microbiota-derived indole metabolites and their regulatory roles in host–tumor interactions. Notably, melanoma has emerged as a key model for integrating tryptophan metabolism with cancer immunotherapy, particularly in clinical trials combining IDO1 inhibitors with PD-1/PD-L1 blockade. Collectively, these findings underscore tryptophan metabolism as a highly conserved and targetable pathway across diverse tumor types, offering a solid theoretical foundation for the development of both broad-spectrum and tumor-specific therapeutic strategies.

To date, the majority of studies have relied on *in vitro* cancer cell lines and *in vivo* murine models, primarily utilizing syngeneic or xenograft systems. Bibliometric analysis reveals that “*in vitro*” and “*in vivo*” were prominent burst keywords during 2015–2017, indicating a research focus heavily oriented toward basic experimental studies during this period. In recent years, however, there has been a marked shift toward clinical relevance, with increasing use of patient-derived materials such as tumor biopsies, plasma samples, and immune profiling data. The emergence of “inhibitors” as a burst keyword between 2020 and 2022 reflects a transition from mechanistic investigation to therapeutic intervention and translational application. Further keyword burst analysis reveals evolving frontiers in this field. High-frequency terms such as “gut microbiota,” “tumor microenvironment,” “aryl hydrocarbon receptor,” and “cancer immunotherapy” suggest a paradigmatic shift from single-pathway metabolic studies toward integrated perspectives that encompass metabolic–immune–microbial interactions. Future research on tryptophan metabolism is expected to place increasing emphasis on multidimensional mechanistic integration, clinical translation, and the advancement of precision oncology.

### 4.2 Mechanisms of tryptophan metabolism in cancer

**Figure 10:**
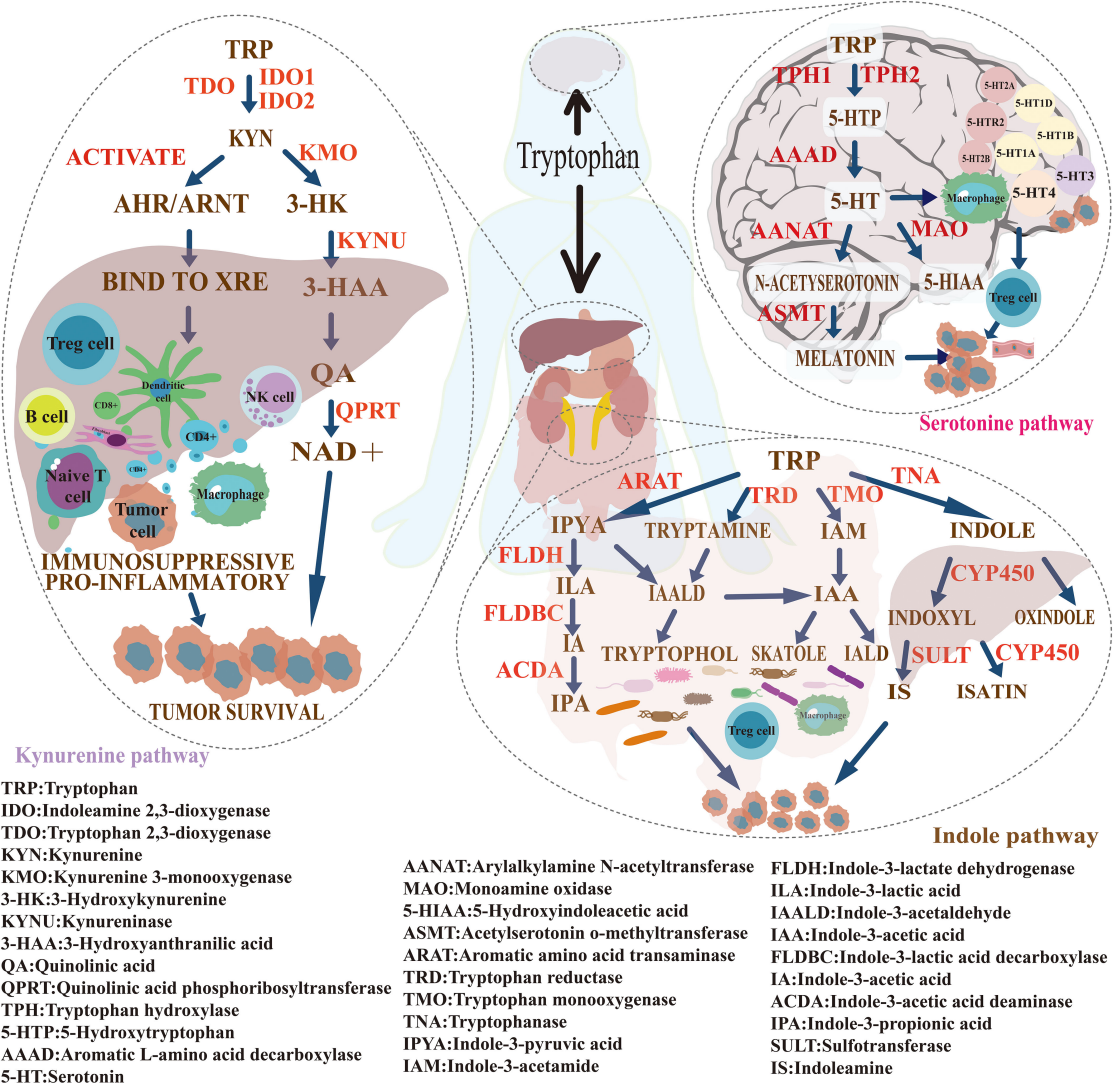
Mechanism illustration of tryptophan metabolism in cancer. Tryptophan is an essential amino acid that must be obtained through dietary intake. Its metabolism primarily occurs via three pathways: the kynurenine (KYN) pathway (approximately 95%), the serotonin (5-HT) pathway (about 1–2%), and the gut microbiota metabolic pathway (around 4–6%). These pathways play important roles in tumorigenesis, immune regulation, and cancer progression.

Tryptophan is an essential amino acid that cannot be synthesized by the human body and must be obtained through dietary intake. Within the human body, tryptophan plays a crucial role in various biological processes, including protein synthesis, neurotransmitter production, and maintaining the normal function of the immune system (37). In recent years, the intricate metabolic pathways and regulatory mechanisms of tryptophan have garnered increasing attention in cancer research. Studies have shown that tryptophan metabolism is closely associated with tumorigenesis and treatment response, and it also serves as a key regulator in the immune evasion process. It modulates malignant phenotypes and contributes to the reprogramming of the tumor immune microenvironment (38). Disruptions in tryptophan metabolism have emerged as critical drivers of cancer progression, through mechanisms involving immune suppression, enhanced cell proliferation, metastasis, and metabolic reprogramming (39). The major metabolic pathways of tryptophan include the kynurenine (KYN) pathway, the serotonin (5-HT) pathway, and the gut microbiota metabolic pathway (40). Among these, the kynurenine pathway accounts for approximately 95% of total tryptophan metabolism, followed by the serotonin pathway (1–2%) and the microbial metabolic pathway (4–6%). **Figure 10** illustrates the mechanisms of tryptophan metabolism in cancer.

#### 4.2.1 Kynurenine pathway

The kynurenine (KYN) pathway, a major route of tryptophan catabolism, plays a pivotal role in tumor immune evasion (41). Bibliometric analysis shows a rising frequency of keywords such as “IDO1,” “AhR,” “inhibitors,” and “kynurenine pathway” in recent years, reflecting sustained interest in this metabolic axis and its translational potential in oncology. In the tumor microenvironment (TME), expression of key enzymes—IDO1 and TDO—is markedly elevated (42). These enzymes convert tryptophan into KYN and downstream metabolites, including quinolinic acid and nicotinamide adenine dinucleotide (NAD^+^), leading to local tryptophan depletion and KYN accumulation (30). Elevated KYN activates the aryl hydrocarbon receptor (AhR), triggering potent immunosuppressive effects. Mechanistically, KYN–AhR signaling suppresses the proliferation and cytotoxicity of effector T cells (CD4^+^, CD8^+^, and CD25^+^) and inhibits the secretion of pro-inflammatory cytokines such as interferon-gamma (IFN-γ) and interleukin-2 (IL-2) (43). At the same time, it promotes the differentiation of naïve CD4^+^ T cells into regulatory T cells (Tregs), increasing the release of immunosuppressive cytokines including transforming growth factor-beta (TGF-β) and interleukin-10 (IL-10) (44). KYN also impairs the activation and receptor expression of natural killer (NK) cells, reducing their antitumor activity. Beyond immunosuppression, KYN and its metabolites activate oncogenic signaling pathways such as MAPK and PI3K/AKT, thereby promoting tumor cell proliferation, survival, epithelial–mesenchymal transition (EMT), and metastatic potential (45).

Given its central role in these processes, IDO1, the rate-limiting enzyme in tryptophan degradation, has emerged as a key therapeutic target in cancer immunotherapy. Among the ten most co-cited references, eight highlight IDO’s role in immune evasion—particularly in cancer—and emphasize the therapeutic promise of IDO inhibitors. The remaining two focus on the role of gut microbiota in modulating immunity and intestinal barrier function via tryptophan metabolism, and on the kynurenine pathway’s relevance as a biomarker and potential target in neuropsychiatric and inflammatory disorders. Collectively, these studies underscore the prominence of “IDO” as a frequently co-occurring keyword, reinforcing its importance in tumor immunology. Preclinical studies have shown that IDO1 inhibitors such as epacadostat can enhance the antitumor efficacy of PD-1 blockade, fueling enthusiasm for targeting this pathway. This enthusiasm is mirrored in bibliometric data, where “IDO” consistently ranks among the top co-occurring terms. However, clinical development has faced significant setbacks. In 2018, the phase III ECHO-301/KEYNOTE-252 trial revealed that combining epacadostat with pembrolizumab failed to improve progression-free survival (PFS) or overall survival (OS) in advanced melanoma patients, raising considerable concern. Similarly, the combination of navoximod with atezolizumab did not achieve its primary efficacy endpoints. These failures have been attributed to issues such as suboptimal dosing, inadequate suppression of intratumoral KYN levels, and potential paradoxical activation of AhR by certain inhibitors, highlighting the complexity of this pathway and the need for deeper mechanistic insights. Consequently, several companies have discontinued related drug development programs (46–48).

Despite these challenges, bibliometric trends indicate a continued—albeit slower—growth in publications, suggesting ongoing interest and a shift in research direction. One emerging focus is the development of predictive biomarkers; for instance, the KYN/Trp ratio is being investigated as a surrogate marker for IDO1 or TDO activity to aid in therapeutic response assessment and patient stratification (49, 50). Another priority is identifying compensatory mechanisms and alternative targets. For example, TDO2 is highly expressed in lung adenocarcinoma, and its inhibition has been shown to downregulate PD-L1 and improve immune responsiveness (51). Kynureninase, an enzyme that degrades KYN and limits AhR activation, has also shown therapeutic potential (52). In parallel, AhR antagonists such as BAY-218 are under investigation as alternatives to IDO1 blockade due to their ability to prevent Treg induction and checkpoint molecule expression (53). Dual inhibitors like RG70099 and CMG017, which simultaneously target IDO1 and TDO2, have demonstrated the ability to reduce systemic KYN levels, offering a strategy to overcome the limitations of monotherapies (54, 55). Synthetic biology approaches are also being explored to integrate IDO1 inhibition with T cell engineering, aiming to enhance antitumor immunity (56). Moreover, noncanonical regulatory mechanisms have attracted attention—for instance, the USP14 inhibitor IU1 has been reported to downregulate IDO1 expression and restore T cell–mediated antitumor responses in colorectal cancer models (57).

#### 4.2.2 5-HT pathways

The 5-hydroxytryptamine (5-HT) metabolic pathway has recently emerged as a crucial regulator in tumor biology, particularly through its involvement in remodeling the tumor microenvironment (TME), mediating immune suppression, and driving metabolic reprogramming. Bibliometric co-occurrence analysis highlights a high frequency of keywords such as “tumor microenvironment,” “immune regulation,” “dendritic cells,” and “metabolic reprogramming,” underscoring the close association between the 5-HT pathway and these cutting-edge research areas. Tumor cells metabolize tryptophan into 5-HT via the enzyme tryptophan hydroxylase 1 (TPH1) (58). Subsequently, 5-HT activates specific receptors, including 5-HT receptor 2B (HTR2B) and 5-HT receptor 7 (HTR7), which initiate signaling cascades that promote metabolic reprogramming and suppress immune responses (59, 60). For instance, 5-HT signaling through 5-HTR2A/C receptors activates Jak1, leading to phosphorylation of STAT3 (61). This event upregulates hypoxia-inducible factor-1α (HIF-1α) and pyruvate kinase M2 (PKM2), thereby enhancing glycolysis and glucose uptake to support rapid tumor proliferation. Concurrently, 5-HT stimulates adenylate cyclase (AC), elevating intracellular cAMP levels, which activate protein kinase A (PKA) and promote phosphorylation of CREB. This pathway improves mitochondrial function, enabling tumor cells to survive under hypoxic and nutrient-deprived conditions (62). By reducing lactate accumulation and tumor acidosis, this cascade also restricts immune cell infiltration. Furthermore, 5-HT activates the PI3K/Akt/mTOR signaling pathway, which further facilitates metabolic reprogramming and immune evasion in the tumor context (63).

Beyond its direct role in metabolic regulation, 5-HT also modulates the tumor immune microenvironment, consistent with bibliometric analyses that highlight “immune regulation” and “dendritic cells” as key research focuses. It promotes the polarization of tumor-associated macrophages (TAMs) toward the M2 phenotype. These M2-type TAMs secrete immunosuppressive cytokines, such as IL-10 and TGF-β, which inhibit the activity of effector T cells and NK cells, thereby facilitating tumor immune evasion (64–66). The immunosuppressive milieu established by M2 TAMs further supports tumor growth and metastasis. In colorectal cancer models, knockout of TPH2 significantly suppresses tumor growth, suggesting that TPH2-positive neurons in the gut promote cancer stem cell (CSC) proliferation through 5-HT secretion (67). Moreover, 5-HT stabilizes β-catenin and stimulates CSC expansion by activating the Wnt/β-catenin signaling pathway, thereby driving tumor progression and metastasis. Its metabolite, 5-hydroxyindoleacetic acid (5-HIAA), enhances neutrophil migration and inflammatory responses via activation of the G protein-coupled receptor 35 (GPR35), which further contributes to immune evasion (68). Additionally, 5-HT functions in an autocrine manner through the HTR2B receptor to increase aerobic glycolysis in tumor cells under metabolic stress, supplying essential substrates for tumor growth (69). This mechanism reinforces the concept of “metabolic reprogramming,” as identified in keyword cluster analyses.

Despite the role of 5-HT metabolites in promoting tumor immune evasion, this pathway’s activity can be effectively modulated through pharmacological interventions. TPH1 inhibitors, such as telotristat ethyl (TE), have received FDA approval for treating carcinoid syndrome and exhibit potent inhibition of 5-HT synthesis (70). Preclinical studies have demonstrated that TE suppresses tumor growth across various cancer types and enhances the antitumor efficacy of immune checkpoint inhibitors. Other TPH inhibitors, including LP-533401 and p-chlorophenylalanine (CPA), have shown antitumor activity in breast cancer and cholangiocarcinoma models (71, 72). These findings correspond with bibliometric data that frequently highlight keywords such as “immune checkpoint” and “inhibitor,” underscoring the translational potential of targeting the 5-HT pathway. Beyond TPH1 inhibition, alternative strategies targeting 5-HT signaling have garnered increasing attention. Selective serotonin reuptake inhibitors (SSRIs), such as fluoxetine and sertraline, have demonstrated antitumor effects in models of breast cancer, colorectal cancer, hepatocellular carcinoma, and glioblastoma (73–76). Their mechanisms of action likely involve induction of apoptosis, inhibition of cell proliferation, and activation of the p53 signaling pathway. Furthermore, both agonists and antagonists of 5-HT receptors show promise in cancer therapy. For example, tropisetron and palonosetron, two 5-HT3 receptor antagonists commonly used to alleviate chemotherapy-induced side effects, have also been found to inhibit the growth and metastasis of colorectal and lung cancers (77). Monoamine oxidase A (MAOA), which is highly expressed in prostate cancer and associated with increased tumor aggressiveness and poor prognosis, represents another therapeutic target. MAOA inhibitors such as clorgyline and phenelzine have demonstrated the ability to slow tumor progression in prostate cancer models and may restore sensitivity to enzalutamide (78, 79). Early clinical evidence further suggests that phenelzine may benefit patients with biochemically recurrent castration-sensitive prostate cancer, and its combination with docetaxel may enhance antitumor efficacy (80).

#### 4.2.3 Indole pathway

Keyword burst analysis highlights a recent surge in terms such as “gut microbiota,” “immune checkpoint blockade,” and “metabolic reprogramming” between 2022 and 2024, reflecting the expanding use of multi-omics technologies in tryptophan metabolism research. Single-cell transcriptomics, metabolomics, and microbiome sequencing are increasingly deployed to dissect how tryptophan-derived metabolites modulate immune dynamics and shape the TME (81). A landmark study by Gao et al. (2018) defined a mechanistic framework linking microbial tryptophan metabolism to immune regulation and epithelial integrity, laying the foundation for the “microbiota–tryptophan–immune axis” (82). Indole derivatives generated by gut microbes—e.g., indole-3-aldehyde (I3A), indole acrylic acid (IA), indole-3-acetic acid (IAA), and indoxyl sulfate (IS)—activate the aryl hydrocarbon receptor (AhR), thereby orchestrating immune cell behavior and TME remodeling (83). Certain metabolites, particularly those linked to indole-3-pyruvate (I3P), drive M2-like polarization of tumor-associated macrophages (TAMs) through AhR signaling, fostering immune suppression and tumor growth (84). Others inhibit indoleamine 2,3-dioxygenase 1 (IDO1), reducing kynurenine (KYN) levels and partially reversing immune evasion (85). Downstream products such as 3-hydroxykynurenine (3-HK) suppress tryptophan hydroxylase 1 (TPH1), lowering serotonin (5-HT) synthesis, which exerts dual immunoregulatory effects (86).

IL4I1, an L-amino acid oxidase, metabolizes tryptophan into I3P and its derivatives. Its elevated expression in immune checkpoint blockade (ICB)-resistant tumors suggests a key role in immunosuppression via AhR activation and positions IL4I1 as a potential therapeutic target (87, 88). This mechanistic insight mirrors bibliometric trends highlighting increased interest in metabolic reprogramming and immune escape. Microbial indole metabolites also show strain-specific immunological consequences. Bacteroides fragilis-derived I3A promotes barrier integrity and immune homeostasis via AhR activation (89), whereas Porphyromonas gingivalis-derived IAA drives immune tolerance and invasive tumor phenotypes (90). These metabolites not only influence local immunity but also reprogram the TME and affect tumor evolution. Synthetic biology provides emerging tools to engineer microbial pathways and therapeutically manipulate tryptophan metabolism (91). Engineered bacteria can be programmed to colonize tumors and deliver immunomodulatory payloads, including cytokines, cytotoxins, or RNA therapeutics. Incorporation of gene circuits—featuring suicide switches, quorum sensing, and logic gates—enhances the safety and precision of these approaches, addressing limitations of conventional therapies (92). Bibliometric trends reflect increasing convergence between microbiome engineering and immunometabolism. Preclinical models show that combining a low-tryptophan diet with IDO1 inhibition produces synergistic anti-tumor effects by limiting Trp availability and suppressing KYN-driven immune evasion. However, microbial compensation via alternative Trp pathways necessitates balancing dietary interventions with microbiome stability (93). Biomarkers like the plasma KYN/Trp ratio have been associated with immunotherapy outcomes, while microbial-derived indoles are being explored as prognostic indicators (49).

### 4.3 Comparative analysis of tryptophan metabolism and other cancer-related metabolic pathways

In recent years, cancer metabolism research has advanced significantly across multiple metabolic pathways. The development of multi-omics metabolomic technologies has greatly improved our ability to elucidate complex interactions among these pathways, facilitating systematic comparative analyses and the development of precise therapeutic strategies. Among these, the metabolic pathways of glutamine, arginine, glucose, and tryptophan have garnered sustained attention. Although no systematic bibliometric analysis has specifically targeted glutamine metabolism in cancer, some studies have explored its association with diabetes. Between 2001 and 2022, a total of 945 relevant publications were identified, showing a steady growth in output since 2007 and reaching a peak around 2017 (94). Arginine metabolism demonstrates significant potential in tumor immunoregulation, particularly through ASS1 downregulation-mediated arginine deprivation therapy (ADT) and its impact on T cell function as well as the expansion of MDSCs and Tregs. These mechanisms have been observed across various cancers, including pancreatic, liver, small-cell lung, and colorectal carcinomas (95). However, systematic bibliometric analyses on this metabolic pathway remain limited. Glucose metabolism, due to its central role in energy metabolic reprogramming, was among the earliest focal points in cancer metabolism research. Taking breast cancer as an example, 957 related publications were identified between 2004 and 2024. The number of publications has risen markedly since 2015, nearly tripling compared to 2010, and peaked in 2022 with over 100 articles, reflecting sustained and active research interest in this field (96). Bibliometric research on tryptophan metabolism is comparatively abundant, with 1,927 relevant publications included from 2005 to 2024. The overall trend demonstrates steady growth, with a pronounced acceleration between 2019 and 2021, culminating in an annual peak of 196 publications in 2021. Although its period of heightened activity began slightly later than that of glutamine metabolism, the peak publication volume notably surpasses those of glutamine and glucose metabolism, reflecting a rapid increase in research interest within this field in recent years.

### 4.4 Limitations

This study presents the first bibliometric analysis that systematically investigates the global research landscape on tryptophan metabolism in cancer from 2005 to 2024. By leveraging multidimensional visualization tools, including CiteSpace, VOSviewer, Python, and R Bibliometrix, this study provides a comprehensive overview of research trends, key contributors, and emerging topics, offering valuable insights into the evolution of this field. Despite its significant academic contributions, this study has certain limitations. First, the dataset is exclusively sourced from the Web of Science Core Collection and only includes English-language publications. This restriction may lead to the exclusion of relevant studies published in other languages, potentially limiting the comprehensiveness of global research insights. Second, discrepancies in algorithmic approaches and metric calculations among different visualization tools may introduce variations in analytical results. Future studies should consider integrating data from multiple databases, incorporating multilingual literature, and adopting diverse analytical methodologies to enhance the robustness and comprehensiveness of bibliometric assessments.

## 5 Conclusions

This study systematically analyzed the research progress on tryptophan metabolism in cancer from 2005 to 2024 using bibliometric methods, highlighting the rapid expansion and growing importance of this research field. Our analysis confirms that tryptophan metabolism—particularly via the kynurenine pathway—plays a pivotal role in tumor progression, immune suppression, and therapeutic resistance. Despite significant advances, several knowledge gaps remain. First, most current studies are preclinical, and the translation of findings into clinical applications remains limited. The precise molecular mechanisms by which different branches of tryptophan metabolism—kynurenine, serotonin, and microbial indole pathways—interact with immune cells in the tumor microenvironment require further elucidation. Second, there is a lack of validated biomarkers for predicting treatment response or guiding patient stratification, especially in the context of immune checkpoint blockade. Third, the therapeutic potential of targeting multiple metabolic pathways simultaneously has yet to be fully explored.

Future research should prioritize translational studies that bridge basic mechanistic findings with clinical outcomes, including the validation of plasma or tissue-based biomarkers such as the KYN/Trp ratio or microbiota-derived metabolites. Additionally, therapeutic strategies that combine metabolic enzyme inhibitors (e.g., IDO1/TDO, IL4I1) with immunotherapy warrant deeper investigation, particularly in resistant tumors. Integrative multi-omics approaches and systems biology tools will be essential for mapping the complex network of tryptophan metabolism and its crosstalk with other oncogenic pathways. Moreover, synthetic biology and engineered microbial therapeutics offer promising platforms for modulating tryptophan metabolism in a tumor-specific manner. International and interdisciplinary collaborations will be crucial in developing precise, multi-targeted interventions aimed at overcoming tumor immune escape and improving patient outcomes.

## Data Availability

The original contributions presented in the study are included in the article/**Supplementary Material**. Further inquiries can be directed to the corresponding authors.

## References

[1] Brown JS Amend SR Austin RH Gatenby RA Hammarlund EU Pienta KJ. Updating the definition of cancer. Mol Cancer Res. (2023) 21:1142–7. doi: 10.1158/1541-7786 PMC10618731 37409952

[2] Chen SM Cao Z Prettner K Kuhn M Yang JT Jiao LR. Estimates and projections of the global economic cost of 29 cancers in 204 countries and territories from 2020 to 2050. JAMA Oncol. (2023) 9:465–72. doi: 10.1001/jamaoncol.2022.7826 PMC9951101 36821107

[3] Bizuayehu HM Ahmed KY Kibret GD Dadi AF Belachew SA Bagade T. Global disparities of cancer and its projected burden in 2050. JAMA Netw Open. (2024) 7:e2443198. doi: 10.1001/jamanetworkopen.2024.43198 39499513 PMC11539015

[4] Kaur R Bhardwaj A Gupta S. Cancer treatment therapies: traditional to modern approaches to combat cancers. Mol Biol Rep. (2023) 50:9663–76. doi: 10.1007/s11033-023-08809-3 37828275

[5] Nazari A Osati P Fakhr SS Faghihkhorasani F Ghanaatian M Faghihkhorasani F. New emerging therapeutic strategies based on manipulation of the redox regulation against therapy resistance in cancer. Antioxid Redox Signaling. (2024). doi: 10.1089/ars.2023.0491 39506926

[6] Nobari SA Doustvandi MA Yaghoubi SM Oskouei SSS Alizadeh E Nour MA. Emerging trends in quantum dot-based photosensitizers for enhanced photodynamic therapy in cancer treatment. J Pharm Invest. (2024) 55:55–90. doi: 10.1007/s40005-024-00698-3

[7] Shamaeizadeh A Beigi A Naghib SM Tajabadi M Rahmanian M Mozafari MR. Smart nanobiomaterials for gene delivery in localized cancer therapy: an overview from emerging materials and devices to clinical applications. Curr Cancer Drug Targets. (2024) 8:1012–42. doi: 10.2174/0115680096288917240404060506 38644713

[8] Zhao YT Sun JQ Xu XL Su J Du YZ. The potential of nanosystems in disrupting adenosine signaling pathways for tumor immunotherapy. Expert Opin Drug Deliv. (2024) 21:1755–70. doi: 10.1080/17425247.2024.2417687 39434697

[9] Liu Y Zhou Q Song SL Tang S. Integrating metabolic reprogramming and metabolic imaging to predict breast cancer therapeutic responses. Trends Endocrinol Metab. (2021) 32:762–75. doi: 10.1016/j.tem.2021.07.001 34340886

[10] Chen Z Li Y Tan B Zhao Q Fan L Li F. Progress and current status of molecule-targeted therapy and drug resistance in gastric cancer. Drugs Today. (2020) 56:469–82. doi: 10.1358/dot.2020.56.7.3112071 32648857

[11] Martins AC Oshiro MY Albericio F de la Torre BG. Food and drug administration (Fda) approvals of biological drugs in 2023. Biomedicines. (2024) 12:1992. doi: 10.3390/biomedicines12091992 39335511 PMC11428688

[12] Yuan S Yu B Liu HM. New drug approvals for 2019: synthesis and clinical applications. Eur J Med Chem. (2020) 205:112667. doi: 10.1016/j.ejmech.2020.112667 32911308

[13] Brown DG Wobst HJ. A decade of fda-approved drugs (2010-2019): trends and future directions. J Med Chem. (2021) 64:2312–38. doi: 10.1021/acs.jmedchem.0c01516 33617254

[14] Mathlouthi S Kuryk L Prygiel M Lupo MG Zasada AA Pesce C. Extracellular vesicles powered cancer immunotherapy: targeted delivery of adenovirus-based cancer vaccine in humanized melanoma model. J Controlled Release. (2024) 376:777–93. doi: 10.1016/j.jconrel.2024.10.057 39481685

[15] Chen QQ Guo XJ Ma WX. Opportunities and challenges of cd47-targeted therapy in cancer immunotherapy. Oncol Res. (2024) 32:49–60. doi: 10.32604/or.2023.042383 PMC10767231 38188674

[16] Joshi DC Sharma A Prasad S Singh K Kumar M Sherawat K. Novel therapeutic agents in clinical trials: emerging approaches in cancer therapy. Discov Oncol. (2024) 15:342. doi: 10.1007/s12672-024-01195-7 39127974 PMC11317456

[17] Meira M Frey A Chekkat N Rybczynska M Sellam Z Park JS. Targeting rgmb interactions: discovery and preclinical characterization of potent anti-rgmb antibodies blocking multiple ligand bindings. Mabs. (2024) 16:2432403. doi: 10.1080/19420862.2024.2432403 39588913 PMC11601088

[18] Wang Y Zhao LW Zhang Z Liu P. Immunogenic cell death inducers and pd-1 blockade as neoadjuvant therapy for rectal cancer. Oncoimmunology. (2024) 13:2416558. doi: 10.1080/2162402x.2024.2416558 39429516 PMC11487966

[19] Arner EN Rathmell JC. Metabolic programming and immune suppression in the tumor microenvironment. Cancer Cell. (2023) 41:421–33. doi: 10.1016/j.ccell.2023.01.009 PMC10023409 36801000

[20] Xue C Li G Zheng Q Gu X Shi Q Su Y. Tryptophan metabolism in health and disease. Cell Metab. (2023) 35:1304–26. doi: 10.1016/j.cmet.2023.06.004 37352864

[21] Zárate LV Miret NV Candia AJN Zappia CD Pontillo CA Chiappini FA. Breast cancer progression and kynurenine pathway enzymes are induced by hexachlorobenzene exposure in a her2-positive model. Food Chem Toxicol. (2023) 177:113822. doi: 10.1016/j.fct.2023.113822 37169060

[22] Perez-Castro L Garcia R Venkateswaran N Barnes S Conacci-Sorrell M. Tryptophan and its metabolites in normal physiology and cancer etiology. FEBS J. (2023) 290:7–27. doi: 10.1111/febs.16245 34687129 PMC9883803

[23] Wu XA Jin B Liu X Mao YL Wan XS Du SD. Research trends of cellular immunotherapy for primary liver cancer: A bibliometric analysis. Hum Vaccines Immunotherapeut. (2024) 20:2426869. doi: 10.1080/21645515.2024.2426869 PMC11572085 39538378

[24] Li JQ Li XX Fu YY Meng HB Xu D Hou WY. Visualizing immunotherapy for multiple myeloma worldwide from 2013 to 2023: A bibliometric analysis. Hum Vaccines Immunotherapeut. (2024) 20:2433304. doi: 10.1080/21645515.2024.2433304 PMC11633138 39639463

[25] Chen YM Lu XJ Peng GY Liu SJ Wang M Hou HM. A bibliometric analysis of research on pd-1/pd-L1 in urinary tract tumors. Hum Vaccines Immunotherapeut. (2024) 20:2390727. doi: 10.1080/21645515.2024.2390727 PMC11469446 39385743

[26] Zheng DT Chen LZ Tian HT Yang QP Wu JY Ji ZQ. A scientometric analysis of research trends on emerging contaminants in the field of cancer in 2012-2021. Front Public Health. (2022) 10:1034585. doi: 10.3389/fpubh.2022.1034585 36504950 PMC9733951

[27] Brandacher G Perathoner A Ladurner R Schneeberger S Obrist P Winkler C. Prognostic Value of Indoleamine 2,3-Dioxygenase Expression in Colorectal Cancer: Effect on Tumor-Infiltrating T Cells. Clin Cancer Res. (2006) 12:1144–51. doi: 10.1158/1078-0432.CCR-05-1966 16489067

[28] Pilotte L Larrieu P Stroobant V Colau D Dolusic E Frederick R. Reversal of Tumoral Immune Resistance by Inhibition of Tryptophan 2,3-Dioxygenase. Proc Natl Acad Sci U S A. (2012) 109:2497–502. doi: 10.1073/pnas.1113873109 22308364 PMC3289319

[29] Munn DH Mellor AL. Indoleamine 2,3-Dioxygenase and Tumor-Induced Tolerance. J Clin Invest. (2007) 117:1147–54. doi: 10.1172/JCI31178 17476344 PMC1857253

[30] Platten M Nollen EAA Röhrig UF Fallarino F Opitz CA. Tryptophan metabolism as a common therapeutic target in cancer, neurodegeneration and beyond. Nat Rev Drug Discov. (2019) 18:379–401. doi: 10.1038/s41573-019-0016-5 30760888

[31] Metz R Duhadaway JB Kamasani U Laury-Kleintop L Muller AJ Prendergast GC. Novel Tryptophan Catabolic Enzyme Ido2 Is the Preferred Biochemical Target of the Antitumor Indoleamine 2,3-Dioxygenase Inhibitory Compound D-1-Methyl-Tryptophan. Cancer Res. (2007) 67:7082–7087. doi: 10.1158/0008-5472.CAN-07-1872 17671174

[32] Platten M Wick W Van den Eynde BJ. Tryptophan Catabolism in Cancer: Beyond Ido and Tryptophan Depletion. Cancer Res. (2012) 72:5435–5440. doi: 10.1158/0008-5472.CAN-12-0569 23090118

[33] Liu X Shin N Koblish HK Yang G Wang Q Wang K. Selective Inhibition of Ido1 Effectively Regulates Mediators of Antitumor Immunity. Blood. (2010) 115:3520–3530. doi: 10.1182/blood-2009-09-246124 20197554

[34] Lob S Konigsrainer A Zieker D Brucher BL Rammensee HG Opelz G. Ido1 and Ido2 Are Expressed in Human Tumors: Levo- but Not Dextro-1-Methyl Tryptophan Inhibits Tryptophan Catabolism. Cancer Immunol Immunother. (2009) 58:153–157. doi: 10.1007/s00262-008-0513-6 18418598 PMC11030193

[35] Sharma MD Baban B Chandler P Hou DY Singh N Yagita H. Plasmacytoid Dendritic Cells from Mouse Tumor-Draining Lymph Nodes Directly Activate Mature Tregs Via Indoleamine 2,3-Dioxygenase. J Clin Invest. (2007) 117:2570–2582. doi: 10.1172/JCI31911 17710230 PMC1940240

[36] Lob S Konigsrainer A Rammensee HG Opelz G Terness P. Inhibitors of Indoleamine-2,3-Dioxygenase for Cancer Therapy: Can We See the Wood for the Trees? Nat Rev Cancer. (2009) 9:445–452. doi: 10.1038/nrc2639 19461669

[37] Zhang HL Zhang AH Miao JH Sun H Yan GL Wu FF. Targeting regulation of tryptophan metabolism for colorectal cancer therapy: A systematic review. Rsc Adv. (2019) 9:3072–80. doi: 10.1039/c8ra08520j PMC9060217 35518968

[38] Zhang SX Chen SL Wang ZH Li JH Yuan YB Feng WT. Prognosis prediction and tumor immune microenvironment characterization based on tryptophan metabolism-related genes signature in brain glioma. Front Pharmacol. (2022) 13:1061597. doi: 10.3389/fphar.2022.1061597 36386216 PMC9663932

[39] Li F Hu HY Li LY Ding LF Lu ZY Mao XD. Integrated machine learning reveals the role of tryptophan metabolism in clear cell renal cell carcinoma and its association with patient prognosis. Biol Direct. (2024) 19:132. doi: 10.1186/s13062-024-00576-w 39707545 PMC11662763

[40] Hou YJ Li J Ying SH. Tryptophan metabolism and gut microbiota: A novel regulatory axis integrating the microbiome, immunity, and cancer. Metabolites. (2023) 13:1166. doi: 10.3390/metabo13111166 37999261 PMC10673612

[41] Tan QW Deng SH Xiong LJ. Role of kynurenine and its derivatives in liver diseases: recent advances and future clinical perspectives. Int J Mol Sci. (2025) 26:968. doi: 10.3390/ijms26030968 39940736 PMC11816720

[42] Seo S-K Kwon B. Immune regulation through tryptophan metabolism. Exp Mol Med. (2023) 55:1371–9. doi: 10.1038/s12276-023-01028-7 PMC10394086 37394584

[43] Yang T Li QQ Liu YM Yang B. T cells in pancreatic cancer stroma: tryptophan metabolism plays an important role in immunoregulation. World J Gastroenterol. (2023) 29:2701–3. doi: 10.3748/wjg.v29.i17.2701 PMC10198057 37213408

[44] Xu XL Yuan HY Lv QJ Wu ZJ Fan WH Liu JJ. Indoleamine 2, 3-dioxygenase regulates the differentiation of T lymphocytes to promote the growth of gastric cancer cells through the pi3k/akt/mtor pathway. Cell Biochem Biophysics. (2024) 83:2289–99. doi: 10.1007/s12013-024-01641-x PMC12089202 39695014

[45] Wang LX Zhou X Yan HS Miao YP Wang BB Gu YH. Deciphering the role of tryptophan metabolism-associated genes echs1 and aldh2 in gastric cancer: implications for tumor immunity and personalized therapy. Front Immunol. (2024) 15:1460308. doi: 10.3389/fimmu.2024.1460308 39328412 PMC11424447

[46] De Martino M Rathmell JC Galluzzi L Vanpouille-Box C. Cancer cell metabolism and antitumour immunity. Nat Rev Immunol. (2024) 24:654–69. doi: 10.1038/s41577-024-01026-4 PMC11365797 38649722

[47] Luke JJ Fakih M Schneider C Chiorean EG Bendell J Kristeleit R. Phase I/ii sequencing study of azacitidine, epacadostat, and pembrolizumab in advanced solid tumors. Br J Cancer. (2023) 128:2227–35. doi: 10.1038/s41416-023-02267-1 PMC10241827 37087488

[48] Lu ZH Zhang CC Zhang J Su W Wang GY Wang ZQ. The kynurenine pathway and indole pathway in tryptophan metabolism influence tumor progression. Cancer Med. (2025) 14:e70703. doi: 10.1002/cam4.70703 40103267 PMC11919716

[49] Mandarano M Orecchini E Bellezza G Vannucci J Ludovini V Baglivo S. Kynurenine/tryptophan ratio as a potential blood-based biomarker in non-small cell lung cancer. Int J Mol Sci. (2021) 22:4403. doi: 10.3390/ijms22094403 33922388 PMC8122814

[50] Meireson A Ferdinande L Haspeslagh M Hennart B Allorge D Ost P. Clinical relevance of serum kyn/trp ratio and basal and ifnγ-upregulated ido1 expression in peripheral monocytes in early stage melanoma. Front Immunol. (2021) 12:736498. doi: 10.3389/fimmu.2021.736498 34557196 PMC8453201

[51] Jin E Yin ZD Zheng XX Yan CH Xu K Eunice FY. Potential of targeting tdo2 as the lung adenocarcinoma treatment. J Proteome Res. (2024) 23:1341–50. doi: 10.1021/acs.jproteome.3c00746 38421152

[52] Labadie BW Bao RY Luke JJ. Reimagining ido pathway inhibition in cancer immunotherapy via downstream focus on the tryptophan-kynurenine-aryl hydrocarbon axis. Clin Cancer Res. (2019) 25:1462–71. doi: 10.1158/1078-0432.Ccr-18-2882 PMC6397695 30377198

[53] Gutcher I Kober C Roese L Roewe J Schmees N Prinz F. Blocking tumor-associated immune suppression with bay-218, a novel, selective aryl hydrocarbon receptor (Ahr) inhibitor. Cancer Res. (2019) 79:Abstract nr 1288. doi: 10.1158/1538-7445.Am2019-1288

[54] Gyulveszi G Fischer C Mirolo M Stern M Green L Ceppi M. Rg70099: A novel, highly potent dual ido1/tdo inhibitor to reverse metabolic suppression of immune cells in the tumor micro-environment. Cancer Res. (2016) 76:Abstract nr LB-085. doi: 10.1158/1538-7445.Am2016-lb-085

[55] Kim C Kim JH Kim JS Chon HJ Kim JH. A novel dual inhibitor of 100 and tdo, cmg017, potently suppresses the kynurenine pathway and overcomes resistance to immune checkpoint inhibitors. J Clin Oncol. (2019) 37:e14228. doi: 10.1200/JCO.2019.37.15_suppl.e14228

[56] Wang H Xu F Yao CL Dai HX Xu JL Wu BB. Engineering bacteria for cancer immunotherapy by inhibiting ido activity and reprogramming cd8+T cell response. Proc Natl Acad Sci United States America. (2024) 121:e2412070121. doi: 10.1073/pnas.2412070121 PMC11670085 39693352

[57] Shi DN Wu XQ Jian YT Wang JY Huang CM Mo S. Usp14 promotes tryptophan metabolism and immune suppression by stabilizing ido1 in colorectal cancer. Nat Commun. (2022) 13:5644. doi: 10.1038/s41467-022-33285-x 36163134 PMC9513055

[58] Siddiqui EJ Thompson CS Mikhailidis DP Mumtaz FH. The role of serotonin in tumour growth (Review). Oncol Rep. (2005) 14:1593–7. doi: 10.3892/or.14.6.1593 16273262

[59] Julius D Livelli TJ Jessell TM Axel R. Ectopic expression of the serotonin 1c receptor and the triggering of Malignant transformation. Science. (1989) 244:1057–62. doi: 10.1126/science.2727693 2727693

[60] Sola-Penna M Paixao LP Branco JR Ochioni AC Albanese JM Mundim DM. Serotonin activates glycolysis and mitochondria biogenesis in human breast cancer cells through activation of the jak1/stat3/erk1/2 and adenylate cyclase/pka, respectively. Br J Cancer. (2020) 122:194–208. doi: 10.1038/s41416-019-0640-1 31819176 PMC7052254

[61] Wehde BL Radler PD Shrestha H Johnson SJ Triplett AA Wagner KU. Janus kinase 1 plays a critical role in mammary cancer progression. Cell Rep. (2018) 25:2192–207 e5. doi: 10.1016/j.celrep.2018.10.063 30463015 PMC6431084

[62] Jiang SH Li J Dong FY Yang JY Liu DJ Yang XM. Increased serotonin signaling contributes to the warburg effect in pancreatic tumor cells under metabolic stress and promotes growth of pancreatic tumors in mice. Gastroenterology. (2017) 153:277–91.e19. doi: 10.1053/j.gastro.2017.03.008 28315323

[63] Zheng YY Li LF Shen ZB Wang LH Niu XY Wei YJ. Mechanisms of neural infiltration-mediated tumor metabolic reprogramming impacting immunotherapy efficacy in non-small cell lung cancer. J Exp Clin Cancer Res. (2024) 43:284. doi: 10.1186/s13046-024-03202-9 39385213 PMC11465581

[64] Ye D Xu HJ Tang QL Xia HW Zhang CL Bi F. The role of 5-ht metabolism in cancer. Biochim Et Biophys Acta-Reviews Cancer. (2021) 1876:188618. doi: 10.1016/j.bbcan.2021.188618 34428515

[65] Yu HF Qu TY Yang JL Dai Q. Serotonin acts through yap to promote cell proliferation: mechanism and implication in colorectal cancer progression. Cell Commun Signaling. (2023) 21:75. doi: 10.1186/s12964-023-01096-2 PMC10100184 37046308

[66] Ge C Yan J Yuan X Xu G. A positive feedback loop between tryptophan hydroxylase 1 and beta-catenin/zbp-89 signaling promotes prostate cancer progression. Front Oncol. (2022) 12:923307. doi: 10.3389/fonc.2022.923307 36172162 PMC9510627

[67] Zhu PP Lu TK Chen ZZ Liu BY Fan DD Li C. 5-hydroxytryptamine produced by enteric serotonergic neurons initiates colorectal cancer stem cell self-renewal and tumorigenesis. Neuron. (2022) 110:2268. doi: 10.1016/j.neuron.2022.04.024 35550066

[68] De Giovanni M Chen HW Li XC Cyster JG. Gpr35 and mediators from platelets and mast cells in neutrophil migration and inflammation. Immunol Rev. (2023) 317:187–202. doi: 10.1111/imr.13194 36928841 PMC10504419

[69] Karmakar S Lal G. Role of serotonin receptor signaling in cancer cells and anti-tumor immunity. Theranostics. (2021) 11:5296–312. doi: 10.7150/thno.55986 PMC8039959 33859748

[70] Schneider MA Heeb L Beffinger MM Pantelyushin S Linecker M Roth L. Attenuation of peripheral serotonin inhibits tumor growth and enhances immune checkpoint blockade therapy in murine tumor models. Sci Trans Med. (2021) 13:eabc8188. doi: 10.1126/scitranslmed.abc8188 34524861

[71] Gwynne WD Hallett RM Girgis-Gabardo A Bojovic B Dvorkin-Gheva A Aarts C. Serotonergic system antagonists target breast tumor initiating cells and synergize with chemotherapy to shrink human breast tumor xenografts. Oncotarget. (2017) 8:32101–16. doi: 10.18632/oncotarget.16646 PMC5458271 28404880

[72] Alpini G Invernizzi P Gaudio E Venter J Kopriva S Bernuzzi F. Serotonin metabolism is dysregulated in cholangiocarcinoma, which has implications for tumor growth. Cancer Res. (2008) 68:9184–93. doi: 10.1158/0008-5472.Can-08-2133 PMC2593938 19010890

[73] Gil-Ad I Zolokov A Lomnitski L Taler M Bar M Luria D. Evaluation of the potential anti-cancer activity of the antidepressant sertraline in human colon cancer cell lines and in colorectal cancer-xenografted mice. Int J Oncol. (2008) 33:277–86. doi: 10.3892/ijo_00000007 18636148

[74] Duarte D Cardoso A Vale N. Synergistic growth inhibition of ht-29 colon and mcf-7 breast cancer cells with simultaneous and sequential combinations of antineoplastics and cns drugs. Int J Mol Sci. (2021) 22:7408. doi: 10.3390/ijms22147408 34299028 PMC8306770

[75] Duarte D Rema A Amorim I Vale N. Drug combinations: A new strategy to extend drug repurposing and epithelial-mesenchymal transition in breast and colon cancer cells. Biomolecules. (2022) 12:190. doi: 10.3390/biom12020190 35204691 PMC8961626

[76] Bhagavathula AS Woolf B Rahmani J Vidyasagar K Tesfaye W. Selective serotonin reuptake inhibitor use and the risk of hepatocellular carcinoma: A systematic review and dose-response analysis of cohort studies with one million participants. Eur J Clin Pharmacol. (2022) 78:547–55. doi: 10.1007/s00228-021-03264-0 35039907

[77] Chen L Huang S Wu X He W Song M. Serotonin signalling in cancer: emerging mechanisms and therapeutic opportunities. Clin Transl Med. (2024) 14:e1750. doi: 10.1002/ctm2.1750 38943041 PMC11213692

[78] Gaur S Gross ME Liao CP Qian B Shih JC. Effect of monoamine oxidase a (Maoa) inhibitors on androgen-sensitive and castration-resistant prostate cancer cells. Prostate. (2019) 79:667–77. doi: 10.1002/pros.23774 PMC7462252 30693539

[79] Wang KL Luo J Yeh SY You BS Meng JL Chang P. The mao inhibitors phenelzine and clorgyline revert enzalutamide resistance in castration resistant prostate cancer. Nat Commun. (2020) 11:2689. doi: 10.1038/s41467-020-15396-5 32483206 PMC7264333

[80] Gross ME Agus DB Dorff TB Pinski JK Quinn D Castellanos O. Phase 2 trial of monoamine oxidase inhibitor phenelzine in biochemical recurrent prostate cancer. Prostate Cancer Prostatic Dis. (2021) 24:61–8. doi: 10.1038/s41391-020-0211-9 PMC7483294 32123315

[81] Rauth S Malafa M Ponnusamy MP Batra SK. Emerging trends in gastrointestinal cancer targeted therapies: harnessing tumor microenvironment, immune factors, and metabolomics insights. Gastroenterology. (2024) 167:867–84. doi: 10.1053/j.gastro.2024.05.005 PMC11793124 38759843

[82] Gao J Xu K Liu H Liu G Bai M Peng C. Impact of the gut microbiota on intestinal immunity mediated by tryptophan metabolism. Front Cell Infect Microbiol. (2018) 8:13. doi: 10.3389/fcimb.2018.00013 29468141 PMC5808205

[83] Hubková B Valko-Rokytovská M Cizmárová B Zábavníková M Mareková M Birková A. Tryptophan: its metabolism along the kynurenine, serotonin, and indole pathway in Malignant melanoma. Int J Mol Sci. (2022) 23:9160. doi: 10.3390/ijms23169160 36012419 PMC9408957

[84] Pan JL Lin Y Liu XY Zhang XZ Liang TB Bai XL. Harnessing amino acid pathways to influence myeloid cell function in tumor immunity. Mol Med. (2025) 31:44. doi: 10.1186/s10020-025-01099-4 39905317 PMC11796060

[85] Fong W Li Q Ji FF Liang W Lau HCH Kang X. Lactobacillus gallinarum-derived metabolites boost anti-pd1 efficacy in colorectal cancer by inhibiting regulatory T cells through modulating ido1/kyn/ahr axis. Gut. (2023) 72:2272–85. doi: 10.1136/gutjnl-2023-329543 PMC10715476 37770127

[86] Wyatt M Greathouse KL. Targeting dietary and microbial tryptophan-indole metabolism as therapeutic approaches to colon cancer. Nutrients. (2021) 13:1189. doi: 10.3390/nu13041189 33916690 PMC8066279

[87] Zuo M Fang J Huang P Liu S Hou P Wang S. Il4i1-catalyzed tryptophan metabolites mediate the anti-inflammatory function of cytokine-primed human muscle stem cells. Cell Death Discov. (2023) 9:269. doi: 10.1038/s41420-023-01568-x 37507432 PMC10382538

[88] Sadik A Somarribas Patterson LF Öztürk S Mohapatra SR Panitz V Secker PF. Il4i1 is a metabolic immune checkpoint that activates the ahr and promotes tumor progression. Cell. (2020) 182:1252–70.e34. doi: 10.1016/j.cell.2020.07.038 32818467

[89] Hu J Chen JW Xu XJ Hou QL Ren J Yan XH. Gut microbiota-derived 3-phenylpropionic acid promotes intestinal epithelial barrier function via ahr signaling. Microbiome. (2023) 11:102. doi: 10.1186/s40168-023-01551-9 37158970 PMC10165798

[90] Ternes D Tsenkova M Pozdeev VI Meyers M Koncina E Atatri S. The gut microbial metabolite formate exacerbates colorectal cancer progression. Nat Metab. (2022) 4:458. doi: 10.1038/s42255-022-00558-0 35437333 PMC9046088

[91] Gurbatri CR Arpaia N Danino T. Engineering bacteria as interactive cancer therapies. Science. (2022) 378:858–63. doi: 10.1126/science.add9667 PMC10584033 36423303

[92] Jiawen Chen JH Sun H. Current developments in the use of engineered bacteria for cancer therapy. Synthetic Biol J. (2023) 4:690–702. doi: 10.12211/2096-8280.2022-062

[93] Bender MJ McPherson AC Phelps CM Pandey SP Laughlin CR Shapira JH. Dietary tryptophan metabolite released by intratumoral lactobacillus reuteri facilitates immune checkpoint inhibitor treatment. Cell. (2023) 186:1846. doi: 10.1016/j.cell.2023.03.011 37028428 PMC10148916

[94] Zhao M Wang K Lin R Mu F Cui J Tao X. Influence of glutamine metabolism on diabetes development:A scientometric review. Heliyon. (2024) 10:e25258. doi: 10.1016/j.heliyon.2024.e25258 38375272 PMC10875382

[95] Feng T Xie F Lyu Y Yu P Chen B Yu J. The arginine metabolism and its deprivation in cancer therapy. Cancer Lett. (2025) 620:217680. doi: 10.1016/j.canlet.2025.217680 40157492

[96] Huang L Zhao W Sun L Niu D Zhu X Jin C. Research progresses and hotspots on glucose metabolic reprogramming in breast cancer: A bibliometric analysis over the past two decades. Front Oncol. (2024) 14:1493996. doi: 10.3389/fonc.2024.1493996 39876898 PMC11772165

