# Supplementary material for: Global research trends in tryptophan metabolism and cancer: a bibliometric and visualization analysis (2005–2024)
Source: Front Oncol. 2025 Jul 1;15:1621666. doi: 10.3389/fonc.2025.1621666 (PMC12259445; doi:10.3389/fonc.2025.1621666)
Supplement: Supplementary file 1 [file DataSheet1.pdf]

Supplementary Materials

Table S1 Annual Publication on Tryptophan Metabolism and Cancer from 1955 to 2004.

Publication Years	Record Count	% of 2,352	Publication Years	Record Count	% of 2,352
2025	83	3.529	1990	5	0.213
2024	199	8.461	1989	6	0.255
2023	171	7.27	1988	2	0.085
2022	185	7.866	1987	2	0.085
2021	195	8.291	1986	2	0.085
2020	153	6.505	1985	1	0.043
2019	122	5.187	1984	1	0.043
2018	111	4.719	1983	1	0.043
2017	110	4.677	1982	1	0.043
2016	100	4.252	1981	1	0.043
2015	88	3.741	1980	1	0.043
2014	87	3.699	1979	5	0.213
2013	82	3.486	1978	3	0.128
2012	69	2.934	1977	3	0.128
2011	74	3.146	1976	6	0.255
2010	50	2.126	1975	3	0.128
2009	59	2.509	1974	2	0.085
2008	48	2.041	1973	4	0.17
2007	43	1.828	1972	3	0.128
2006	45	1.913	1971	2	0.085
2005	30	1.276	1970	2	0.085
2004	20	0.85	1969	3	0.128
2003	19	0.808	1968	1	0.043
2002	15	0.638	1967	4	0.17
2001	9	0.383	1966	5	0.213
2000	14	0.595	1965	3	0.128
1999	11	0.468	1964	8	0.34
1998	5	0.213	1963	5	0.213
1997	7	0.298	1962	2	0.085
1996	9	0.383	1961	3	0.128
1995	12	0.51	1960	1	0.043
1994	7	0.298	1959	2	0.085
1993	9	0.383	1956	1	0.043
1992	8	0.34	1955	1	0.043
1991	8	0.34			

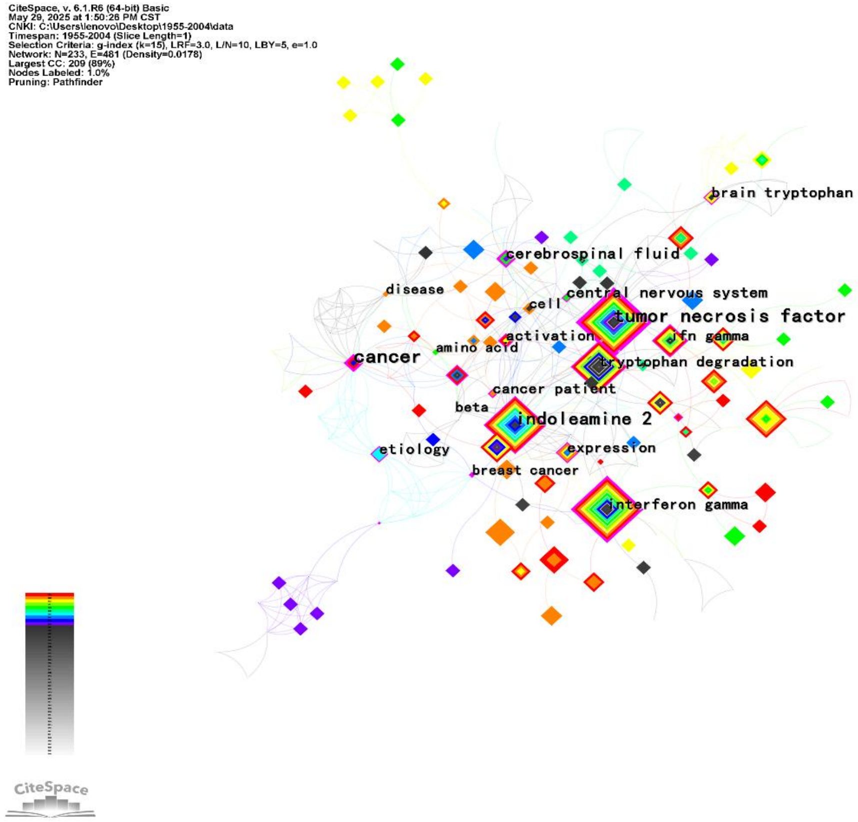

Figure S1 Visualization of the most frequent keywords in research on tryptophan metabolism and cancer (1955–2004)

Table S2 Most frequent keywords related to tryptophan metabolism and cancer (1955–2004).

Rank	Keywords	Count	Centrality	Year
1	interferon gamma	40	0.21	1990
2	tumor necrosis factor	28	0.44	1991
3	indoleamine 2	28	0.19	1990
4	tryptophan catabolism	25	0.09	2000
5	tryptophan degradation	21	0.17	1991
6	activation	12	0.16	1993
7	induction	12	0.08	1991
8	nitric oxide synthase	11	0.06	1999
9	cancer	10	0.27	1990
10	mechanism	10	0.04	1991
11	inhibition	10	0.03	2001

Note: From 1955 to 2004, the annual publication volume remained relatively low, indicating that the field was still in its formative stage. During this period, research primarily explored the biochemical pathways of tryptophan metabolism and the enzymatic activities involved. Although studies were limited in number, several pioneering works began to investigate the immunological implications of tryptophan catabolism, such as the induction of indoleamine 2,3-dioxygenase (IDO) by interferon-gamma, laying the groundwork for later studies on cancer-related

mechanisms.

Table S3 Top 10 most cited publications related to tryptophan metabolism and cancer (1955–2004).

Rank	References	Total Citations	Year
1	Evidence for a tumoral immune resistance mechanism based on tryptophan degradation by indoleamine 2,3-dioxygenase	1877	2003
2	IDO expression by dendritic cells: Tolerance and tryptophan catabolism	1867	2004
3	Inhibition of T cell proliferation by macrophage tryptophan catabolism	1322	1999
4	Relationship between interferon-gamma, indoleamine2,3-dioxygenase, and tryptophan catabolism	928	1991
5	Evidence for an immune-response in major depression - A review and hypothesis	820	1995
6	Expression of indoleamine 2,3-dioxygenase by plasmacytoid dendritic cells in tumor-draining lymph nodes	738	2004
7	Cytokines and psychopathology: Lessons from interferon-α	443	2004
8	Mechanism of interferon-gamma action-characterization of indoleamine 2,3-dioxygenase in cultured human-cells induced by interferon-gamma and evaluation of the enzyme-mediated tryptophan degradation in its anticellular activity	438	1988
9	Interferon-alpha-induced changes in tryptophan metabolism: Relationship to depression and paroxetine treatment	377	2003
10	Enumeration of human colonic bacteria producing phenolic and indolic compounds: Effects of pH, carbohydrate availability and retention time on dissimilatory aromatic amino acid metabolism	377	1996

We conducted a focused analysis of the top three most cited publications from 1955 to 2004, detailed as follows:

Evidence for a Tumoral Immune Resistance Mechanism Based on Tryptophan Degradation by Indoleamine 2,3-Dioxygenase revealed that constitutive expression of indoleamine 2,3-dioxygenase (IDO) in human tumors contributes to immune escape by suppressing the local accumulation of tumor-specific T lymphocytes. The authors further proposed that pharmacological inhibition of IDO could enhance the efficacy of therapeutic cancer vaccines by restoring antitumor immune responses.

The review article *IDO expression by dendritic cells: tolerance and tryptophan catabolism* systematically delineates the pivotal role of indoleamine 2,3-dioxygenase (IDO) in tumor immune evasion and immune tolerance. IDO catalyzes the degradation of tryptophan, thereby restricting T cell proliferation and activation, which facilitates the establishment of an immunosuppressive microenvironment. The article comprehensively summarizes the immunoregulatory functions of IDO across

various contexts, including cancer, pregnancy, chronic infections, and autoimmune diseases. Furthermore, it proposes an integrated model elucidating how IDO-mediated metabolic pathways induce immune tolerance through nutrient depletion and the generation of immunomodulatory metabolites. This review provides critical theoretical insights underpinning IDO-targeted immunotherapeutic strategies and underscores their significant translational potential in oncology.

Inhibition of T cell proliferation by macrophage tryptophan catabolism demonstrates that macrophages induce the expression of indoleamine 2,3-dioxygenase (IDO) to mediate tryptophan catabolism, leading to proliferative arrest of T cells at the G1 phase and thus regulating immune tolerance. The expression of IDO is triggered by IFN-γ and CD40L stimulation, and T cells can only resume proliferation when tryptophan is available alongside a secondary T cell receptor (TCR) signal. This study elucidates, for the first time, the molecular mechanism through which macrophage-expressed IDO controls T cell proliferation, highlighting its critical role in immune regulation and tumor immune evasion, and identifying IDO as a potential target for cancer immunotherapy.

These three seminal publications collectively highlight the critical role of indoleamine 2,3-dioxygenase (IDO) in tumor immune evasion and immune regulation, yet each approaches the topic from distinct perspectives that together form a comprehensive understanding of IDO's function. While all three papers underscore IDO's pivotal role in creating an immunosuppressive microenvironment and identify it as a promising target for cancer immunotherapy, they differ in their emphasis on the cellular sources of IDO (tumor cells, dendritic cells, macrophages) and the mechanistic depth—ranging from tumor microenvironment effects to detailed cell cycle regulation. Together, these studies provide complementary insights that have laid the foundational knowledge for the development of IDO-targeted therapies, highlighting the complexity of tryptophan metabolism's influence on tumor immunity and offering multiple avenues for therapeutic intervention.
